# Oxytocin‐Mediate Modulation of Splenic Immunosuppression in Chronic Social Stress Through Neuroendocrine Pathways

**DOI:** 10.1002/advs.202500849

**Published:** 2025-04-26

**Authors:** Yi‐Shu Zhang, Hai‐Chao Chen, Jia‐Xin Cao, Si‐Wei Zhou, Yue‐Zhang Ma, Yu‐Hong Jing

**Affiliations:** ^1^ Institute of Anatomy and Histology & Embryology Neuroscience School of Basic Medical Sciences Lanzhou University Lanzhou Gansu 730000 P. R. China; ^2^ Department of Immunization Program Shaanxi Provincial Center for Disease Control and Prevention Xi'an P. R. China; ^3^ Key Laboratory of Preclinical Study for New Drugs of Gansu province Lanzhou University Lanzhou Gansu 730000 P. R. China

**Keywords:** brain‐spleen interaction, chronic social stress, immune regulation, macrophage polarization, oxytocin

## Abstract

Chronic social stress (CSS) is a significant public health challenge that negatively impacts behavior and immune function through brain‐spleen interactions. Oxytocin (OT), a neuropeptide critical for social behavior and immune regulation, is upregulated during CSS, though its underlying mechanisms remain unclear. This study investigates the role of OT in splenic immune modulation using a murine model of CSS. Behavioral evaluations, serum oxytocin quantification, and splenic immunophenotypic analysis were performed. Splenic denervation confirmed OT’s neuromodulatory role, whereas OTR antagonism revealed its endocrine function. CSS‐induced OT elevation was associated with immunosuppression, characterized by increased Foxp3⁺ regulatory T cells and reduced CD4⁺ T and CD19⁺ B cells. OT also modulated macrophage polarization, inhibiting M1‐like (pro‐inflammatory) and enhancing M2‐like (anti‐inflammatory) phenotypes. Denervation or pharmacological blockade of OT signaling partly reversed CSS‐induced splenic immunosuppression but adversely affected survival in CSS‐exposed mice. Additionally, denervation or OTR antagonism reduced the mice's response to social defeat, as shown by decreased social avoidance behavior. These findings suggest that OT‐mediated immunosuppression likely represents a compensatory mechanism in response to chronic social stress. Targeting the OT–immune axis could offer innovative therapeutic approaches for stress‐associated disorders by restoring immune homeostasis while maintaining behavioral integrity.

## Graphical abstract

Chronic social stress elevates oxytocin levels, leading to immunosuppression by enhancing regulatory T‐cell activity and promoting anti‐inflammatory macrophage polarization in the spleen. Blocking oxytocin signaling reverses these effects but worsens stress‐induced behavioral disorders and reduces survival rates. This highlights oxytocin's dual role in balancing immune responses and protecting against stress‐related disorders via neuroendocrine pathways.

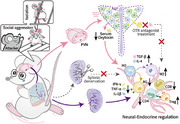

## 1 Introduction

In contemporary society, individuals are increasingly subjected to a range of social pressures. The epidemiological link between elevated stress levels and disease incidence underscores the intricate interplay between mental and physiological health within modern medicine.^[^ ^1^, ^2^ ^]^ Prolonged or recurrent adverse social experiences, such as social exclusion, failure, and perceived threats, can result in Chronic Social Stress (CSS).^[^ ^3^, ^4^ ^]^ In this study, we employed a standardized CSS model using large and aggressive Kunming mice (KM) as the aggressors against C57BL/6 mice. Mice in the CSS group were subjected to 10 min of social aggression daily, followed by confinement in an isolation cage where they had only visual, olfactory, and auditory contact with the aggressor mice for 24 h. This prolonged psychological stress alters the bidirectional communication between the brain and the immune system, contributing to immune dysregulation in many stress‐related psychiatric disorders.^[^ ^5^, ^6^, ^7^ ^]^ Oxytocin (OT), a critical neuropeptide, has garnered significant attention for its roles in modulating social behavior, stress responses, and immune functions.^[^ ^8^, ^9^ ^]^ Although it is established that OT neurons project extensively throughout the brain and that Oxytocin Receptors (OTRs) are widely distributed in peripheral organs,^[^ ^10^, ^11^, ^12^ ^]^ the extent to which brain activity directly modulates immune response via OT neuronal projections remains unclear.

The spleen, a major lymphatic organ involved in immune responses to psychological stress, plays a key role in monocytosis through granulocyte aggregation and ectopic bone marrow formation, leading to elevated circulating monocyte levels.^[^ ^13^, ^14^ ^]^ Research into the brain‐spleen axis has identified it as a direct neural pathway regulating cognition and emotion through peripheral immune response during stress. Studies have shown that activation of CRH^+^ neurons in the PVN and central amygdala (CeA) can appropriately increase the production of splenic plasma cells.^[^ ^14^, ^15^ ^]^ Conversely, disruption of these neural connections reduces plasma cell numbers by lowering glucocorticoid production via the hypothalamic‐pituitary‐adrenal (HPA) axis.^[^ ^16^ ^]^ This finding suggests that immune cells, distributed throughout various tissues, are dynamically regulated by nervous system activity. Investigating potential interactions between OT neurons and the brain‐spleen axis could provide novel insights into the immunoregulatory role of oxytocin.

Research on OT in CSS has primarily focused on its effects on maladaptive behaviors in mice, such as social avoidance, depression, and anxiety, which are associated with dysregulated OT expression.^[^ ^17^, ^18^, ^19^, ^20^ ^]^ OT, acting through OTR, modulates synaptic plasticity and reduces central sensitization in chronic migraine models.^[^ ^21^, ^22^ ^]^ Additionally, OT has demonstrated immunomodulatory effects, including inhibition of lipopolysaccharide (LPS)‐induced microglial activation and reduced release of pro‐inflammatory factors.^[^ ^23^ ^]^ It also enhances CD8^+^ T cell efficacy against tumor cells.^[^ ^24^, ^25^ ^]^ However, OT and OTR functions vary across brain regions and cell types, displaying a high degree of spatial and temporal specificity.^[^ ^26^ ^]^ Investigating the roles of OT and OTR in the spleen is crucial for understanding the complex functions of oxytocin in immune regulation.

## 2 Results

### 2.1 Behavioral and Physiological Impact of Chronic Social Stress in Mice

**Figure 1:**
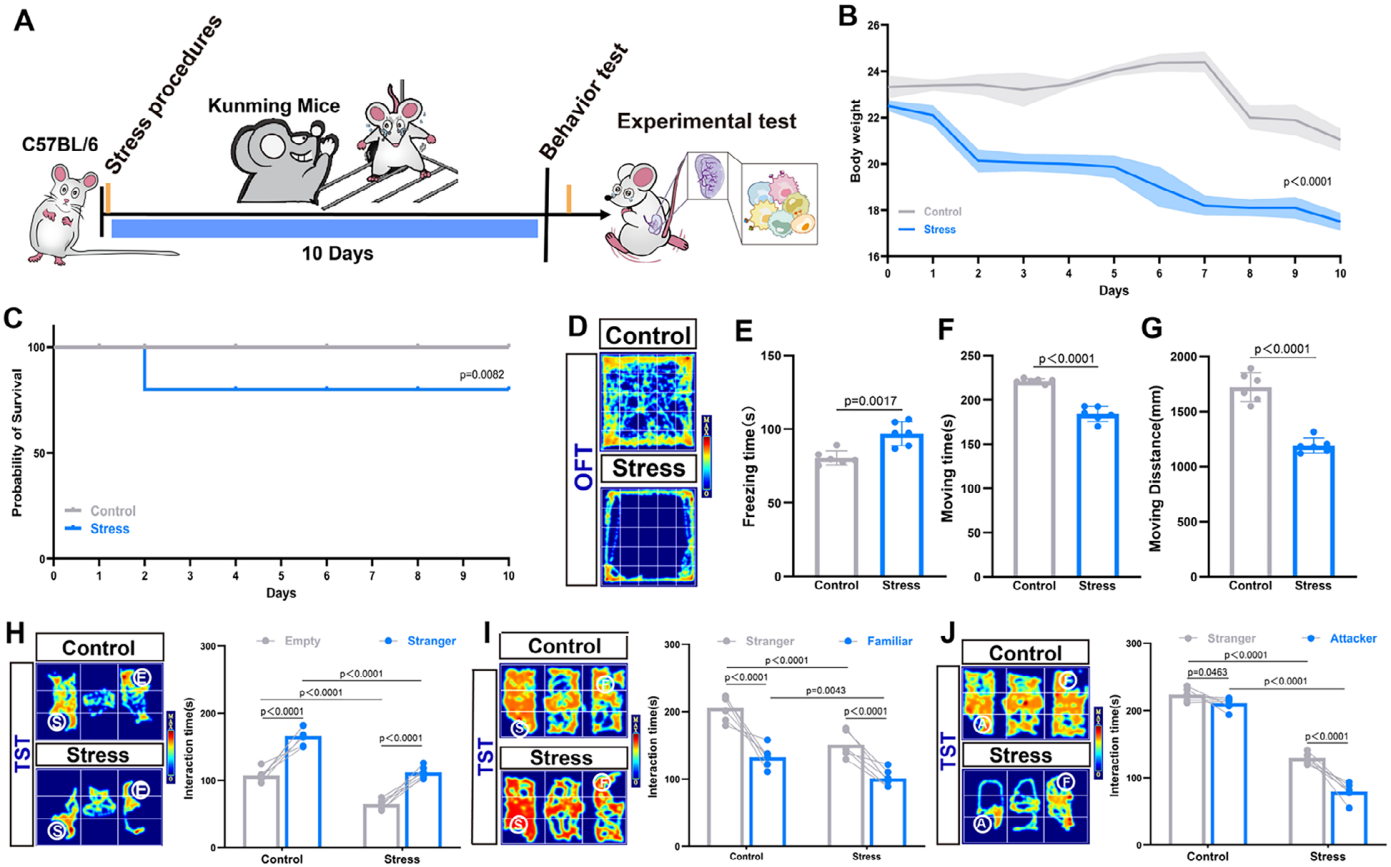
Chronic social stress affects mice's behavior. A) Schematic diagram of the experimental model; B) Changes in mice body weight; C) Variations in mice survival rates; D–G) Results of the OFT; (D) Activity trajectory heatmap; (E) Statistical chart of freezing time; (F) Statistical chart of moving time; (G) Statistical chart of moving distance; H) Social preference in the TST between an empty cage and a stranger mice; I) Social preference in the TST between stranger mice and familiar mice; J) Social preference in the TST between a familiar mice and an attacker mice. One‐way ANOVA and post hoc analyses (E, F, G), Two‐way ANOVA and post hoc analyses (H, I, J), *n* = 6 mice per group.

To investigate the effects of CSS on mice, we established a CSS model (**Figure** **1**A). Body weight in the Stress group decreased significantly compared to pre‐CSS levels, while no significant changes were observed in the Control group (Figure 1B). Additionally, Stress group mice exhibited a significantly reduced survival rate (Figure 1C). H&E staining at the end of the CSS period showed no structural abnormalities in the spleen (Figure S1, Supporting Information). Behavioral responses were assessed using the Open Field Test (OFT) and the Three‐Chamber Social Test (TST). In the OFT, stressed mice demonstrated reduced activity in the central zone, increased freezing duration, and decreased overall locomotion, indicating diminished spontaneous exploration and elevated anxiety levels (Figure 1D–G). The TST indicated that CSS did not impair social ability, preference, or memory, as Stress mice interacted more with other mice than with empty cages, preferred strangers over familiar mice, and chose familiar over attacker mice (Figure 1H–J). Notably, the Control mice, lacking CSS exposure, showed social avoidance toward aggressive mice likely due to their physical presence rather than fearful memories. In summary, CSS significantly increased anxiety levels and reduced spontaneous exploratory behavior in mice, while preserving their social abilities, preferences, and memory.

### 2.2 Chronic Social Stress Alters the Molecular Profiling of the Mice Spleen

**Figure 2:**
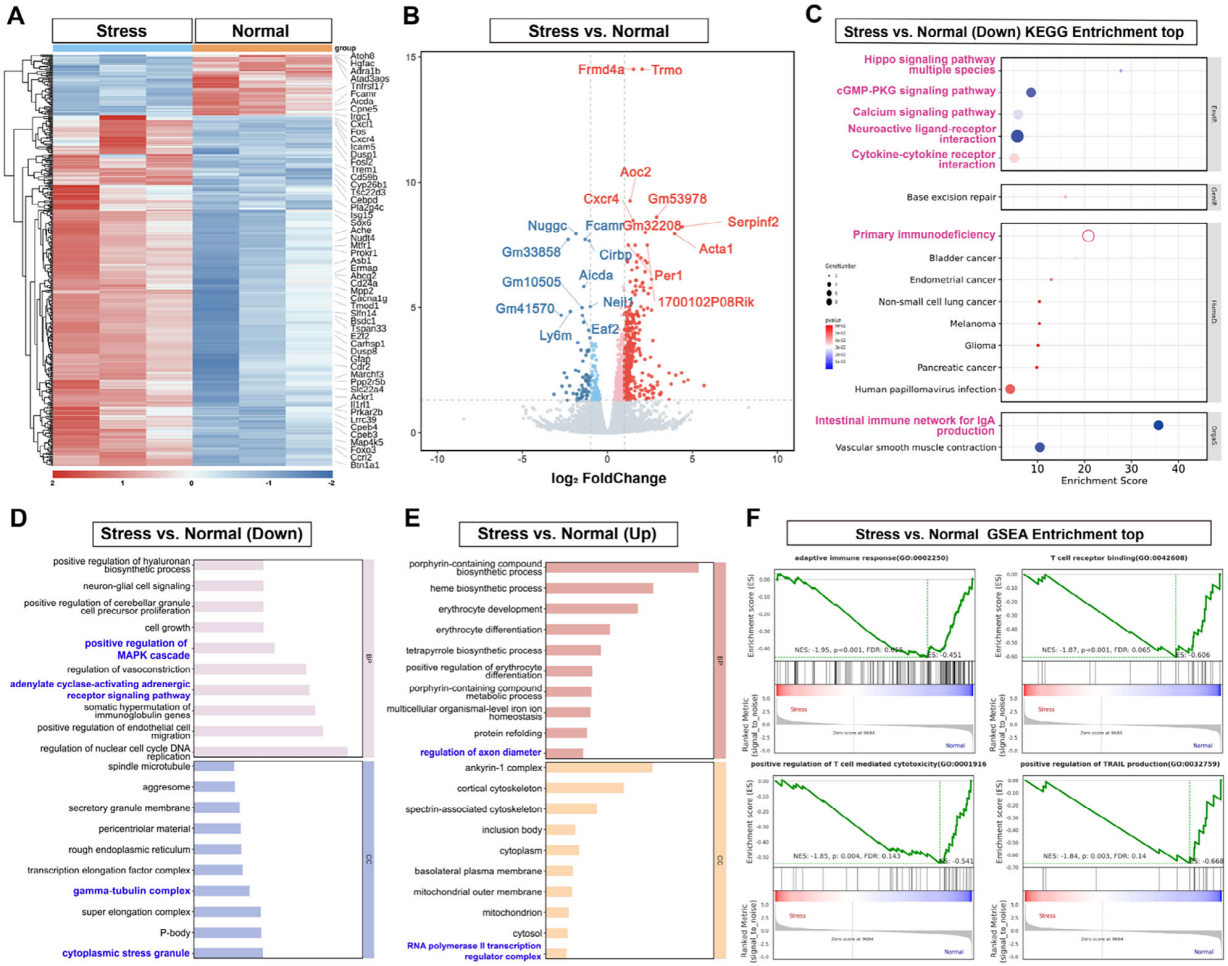
Transcriptional Changes in the Spleen Following CSS. A) Differential Gene Clustering Analysis Heatmap, with the chronic social stress group (left) and the control group (right); B) Volcano plot of differentially expressed genes in the CSS group, with significantly upregulated genes in red (log2 fold change >2 and adjusted *p*‐value < 0.05) and significantly downregulated genes in blue (log2 fold change < −2 and adjusted *p*‐value < 0.05). Wald test and Benjamini–Hochberg correction; C–E) KEGG Enrichment Analysis of Significantly Downregulated Genes in the CSS Group Compared to the Control Group (C), and GO Enrichment Analysis of Significantly Upregulated (D) and Downregulated Genes (E). Bar graphs display the top 10 enriched GO/KEGG terms for biological processes, ranked by FDR‐adjusted p‐values (using g: SCS correction, with a significance threshold of 0.05); F) GSEA plots reveal downregulated GO pathways in the spleens of CSS‐treated mice compared to the control group. GSEA defines significance as *p*‐value < 0.05 and FDR *q*‐value < 0.25; *n* = 3.

To investigate the effects of CSS on splenic function, we analyzed the transcriptional changes in the spleen. Following CSS, we observed significant alterations in splenic gene expression, with 66 genes downregulated and 380 genes upregulated (**Figure** **2**A). Notably, genes associated with immune modulation and inflammation (e.g., Dusp1, Lrrc39, E2f2), neuroplasticity and memory (e.g., Cpeb3, Efnb3), and behavioral disorders (e.g., Pla2g4c) exhibited substantial changes. Volcano plot analysis revealed that genes such as Frmd4a and Cxcr4 were upregulated (Figure 2B). Previous studies have shown that Frmd4a is linked to M2‐like macrophage polarization and Cxcr4 is essential for Treg cell proliferation and activation.^[^ ^27^, ^28^ ^]^ Meanwhile, genes such as Nuggc and Aicda were downregulated (Figure 2B). These genes are associated with B cell differentiation and antibody production.^[^ ^29^ ^]^ KEGG pathway analysis revealed the downregulation of immune‐related pathways, including Neuroactive ligand‐receptor interaction and cGMP‐PKG signaling (Figure 2C). Gene Ontology (GO) analysis following CSS indicated a significant downregulation of pathways related to immune cell proliferation and function, such as “positive regulation of MAPK cascade” and “cytoplasmic stress granule” (Figure 2D), as well as those associated with neuronal function and Treg cell development (Figure 2E). Gene Set Enrichment Analysis (GSEA) further identified the downregulation of key pathways, including “adaptive immune response,” ‘T cell receptor binding,’ “positive regulation of T cell‐mediated cytotoxicity,” and “positive regulation of TRAIL production” (Figure 2F). These findings suggest that CSS impairs splenic immune function in mice, likely through widespread changes in gene expression that affect immune cell activity.

### 2.3 Chronic Social Stress Leads to Splenic Immunosuppression

**Figure 3:**
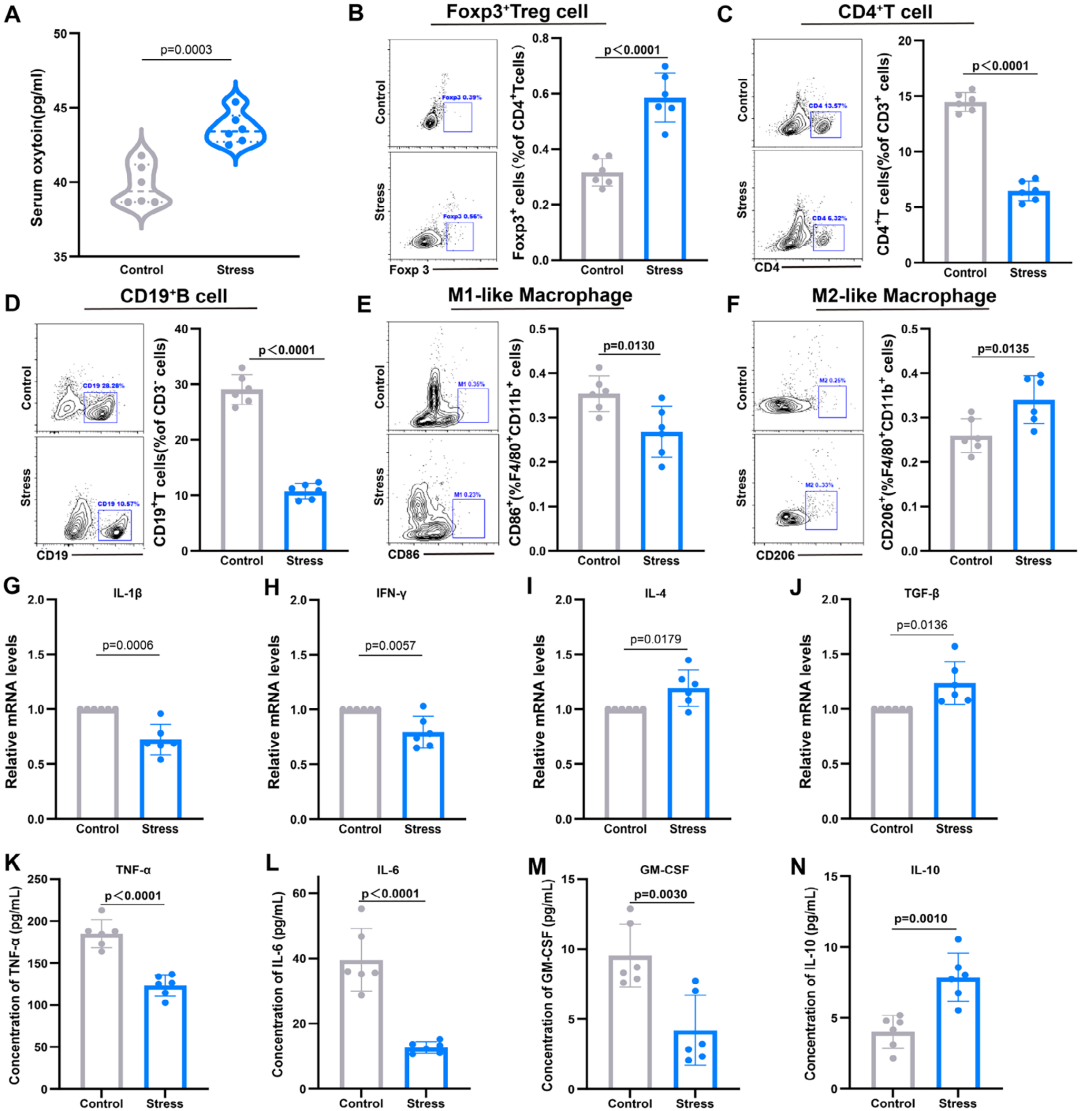
Changes in splenic immune profile and cytokine expression in mice under CSS. A) Changes in serum OT levels in mice; B–F) Representative flow cytometry plots and percentage statistics of CD4^+^ T cells, CD19^+^ B cells, Foxp3^+^ Tregs, F4/80^+^CD11b^+^CD86^+^ M1‐like macrophages, and F4/80^+^CD11b^+^CD206^+^ M2‐like macrophages in the spleens of the Control group and the Stress group; G–J) Relative expression levels of IL‐1β, IFN‐γ, IL‐4, and TGF‐β in the spleens of the Control group and the Stress group, with the control group levels normalized to a value of 1; K–N) The expression levels of TNF‐α, IL‐6, GM‐CSF, and IL‐10 in mouse plasma were detected by ELISA. One‐way ANOVA and post hoc analyses, *n* = 6 mice per group.

To validate transcriptomic findings, we examined changes in splenic immune cells following CSS. CSS resulted in elevated serum OT levels (**Figure** **3**A) and a significant increase in the proportion of Foxp3^+^ Treg cells, accompanied by marked reductions in CD4^+^ T cells and CD19^+^ B cells (Figure 3B–D). Macrophage polarization analysis revealed a shift from pro‐inflammatory F4/80^+^CD11b^+^CD86^+^ M1‐like macrophages to anti‐inflammatory F4/80^+^CD11b^+^CD206^+^ M2‐like macrophages (Figure 3E,F). These findings suggest that OT may contribute to immunosuppressive effects by promoting Treg cell proliferation and influencing macrophage polarization. Further analysis of Treg cell suppressive functions indicated decreased expression of pro‐inflammatory cytokines IL‐1β and IFN‐γ (Figure 3G,H) and increased expression of anti‐inflammatory cytokines IL‐4 and TGF‐β (Figure 3I,J). This cytokine profile implies that Treg cells inhibit CD4^+^ T and CD19^+^ B cell proliferation, likely through TGF‐β secretion, and drive macrophage polarization toward M2‐like phenotypes while suppressing M1‐like polarization by reducing IL‐1β levels. Collectively, the observed elevation of TGF‐β and IL‐4, alongside reduced IL‐1β and IFN‐γ, underscores a state of immunosuppression in the spleen following CSS. We detected the expression levels of TNF‐α, IL‐6, GM‐CSF, and IL‐10 in mouse plasma using ELISA. The results showed that after CSS, TNF‐α, IL‐6, and GM‐CSF levels decreased, while IL‐10 levels increased (Figure 3K–N). These findings provide mechanistic insights into the immunomodulatory effects of CSS and its potential mediation through OT and Treg cells.

### 2.4 Effects of OTR Antagonism on Splenic Immune Responses Following Chronic Social Stress

**Figure 4:**
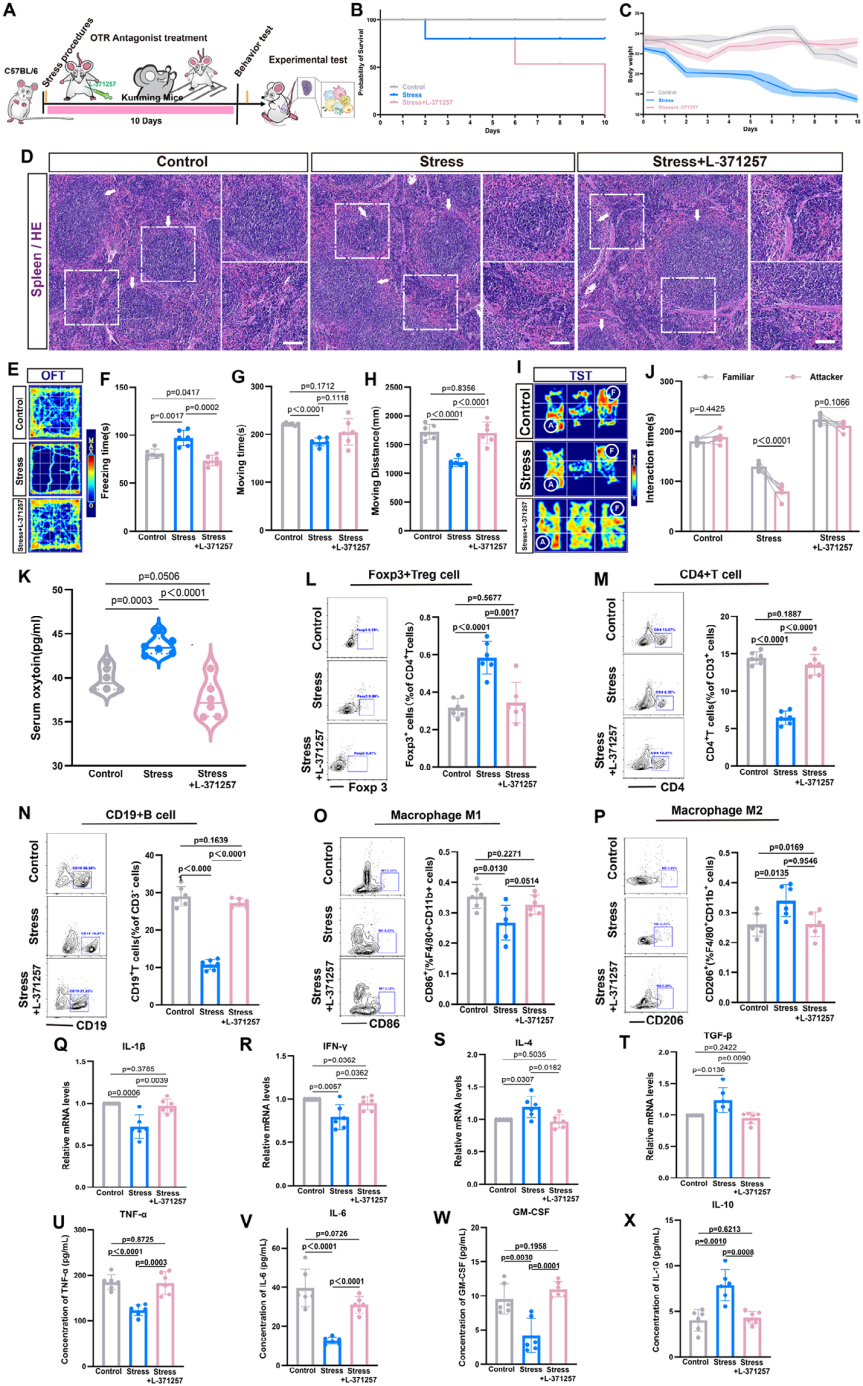
Effects of CSS and L‐371257 treatment on behavior, immune function, and cytokine expression in mice. A) Schematic diagram of the experimental model; B) Changes in mice body weight; C) Variations in mice survival rates; D) Structure of the spleen in H&E‐stained mice; E–H) Results of the OFT; (E) Activity trajectory heatmap; (F) Statistical chart of freezing time; (G) Statistical chart of moving time; (H) Statistical chart of moving distance; I,J) Social preference in the TST between familiar mice and attacker mice; K) Changes in serum OT levels in mice; L–P) Representative flow cytometry plots and percentage statistics of CD4^+^ T cells, CD19^+^ B cells, Foxp3^+^ Tregs, F4/80^+^CD11b^+^CD86^+^ M1‐like macrophages, and F4/80^+^CD11b^+^CD206^+^ M2‐like macrophages in the spleens of the Control group, the Stress group and the Stress+L‐371257 group; Q–T) Relative expression levels of IL‐1β, IFN‐γ, IL‐4, and TGF‐β in the spleens of the Control group, the Stress group, and the Stress+L‐371257 group, with the control group levels normalized to a value of 1; U–X) The expression levels of TNF‐α, IL‐6, GM‐CSF, and IL‐10 in mouse plasma were detected by ELISA. Two‐way ANOVA and post hoc analyses, *n* = 6 mice per group.

In order to investigate the role of OT in modulating the splenic immune response under CSS via the endocrine pathway, we administered the OT receptor (OTR) antagonist L‐371257 intraperitoneally 30 min prior to each social attack (**Figure** **4**A). OTR antagonism significantly altered the physiological and behavioral outcomes of CSS. Mice receiving OTR antagonists exhibited no significant weight loss but showed a markedly reduced survival rate compared to the Stress group. Histological analysis revealed no abnormalities in splenic structure (Figure 4B–D). In OFT, OTR antagonistic mice had enhanced autonomous exploration, including increased walking ability and walking distance, compared with the stress‐responsive group (Figure 4E–H). In the TST, their social abilities and preferences between an empty cage and a stranger mouse, as well as between a stranger and a familiar mouse, remained unaffected (Figure S2, Supporting Information). However, OTR‐antagonized mice lacked social avoidance of aggressive mice and occasionally initiated attacks, suggesting that OT signaling is critical for recognizing and responding to stressors (Figure 4I,J). Serum OT levels were significantly decreased in OTR‐antagonized mice compared to the Stress group (Figure 4K). Immune cell analysis revealed a reduction in Foxp3^+^ Treg cell proportions and increases in CD4^+^ T and CD19^+^ B cell populations in the spleens of OTR‐antagonized mice (Figure 4L–N). Macrophage polarization shifted toward a pro‐inflammatory phenotype, with increased F4/80^+^CD11b^+^CD86^+^ M1‐like macrophages and reduced F4/80^+^CD11b^+^CD206^+^ M2‐like macrophages (Figure 4O,P). Correspondingly, cytokine analysis indicated elevated levels of pro‐inflammatory IL‐1β and IFN‐γ and reduced levels of anti‐inflammatory IL‐4 and TGF‐β (Figure 4Q–T). The expression levels of TNF‐α, IL‐6, GM‐CSF, and IL‐10 in mouse plasma were detected by ELISA. The results showed that, compared with the stress group, after OTR antagonism, TNF‐α, IL‐6, and GM‐CSF levels increased, while IL‐10 levels decreased (Figure 4U–X). Those results suggest that OTR antagonism effectively mitigates CSS‐induced immunosuppression by restoring pro‐inflammatory cytokine production and macrophage polarization. However, the accompanying reduction in survival rates highlights the potential detrimental effects of excessive inflammation. These findings underscore the dual role of OT in balancing immune responses and behavioral adaptation under stress.

### 2.5 Cellular Mechanisms of OT Modulation of Splenic Immune Responses Under Inflammatory Conditions

**Figure 5:**
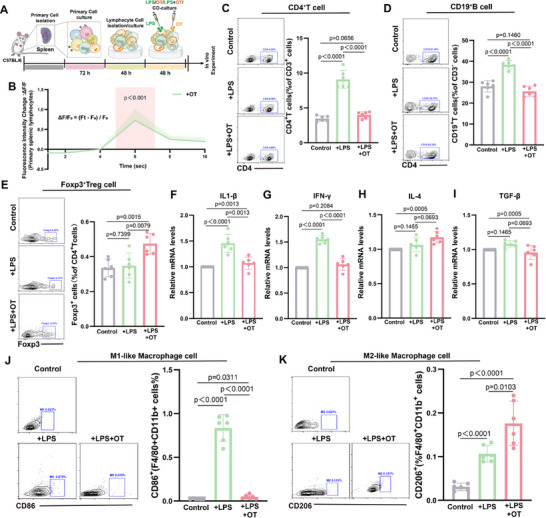
Oxytocin Regulation of Immune Cells. A) Experimental model diagram; B) Changes in cellular calcium signaling after addition of OT. Fluorescence Intensity Change (ΔF/F₀) represents the relative change in fluorescence signal, calculated as ΔF/F₀ = (F_t_ – F₀) / F₀. F_t_ is the fluorescence intensity at a specific time point, and F₀ is the baseline fluorescence intensity (usually the average fluorescence before stimulation); C–E) Representative flow cytometry plots and percentage statistics of Foxp3^+^ Tregs, CD4^+^ T cells, and CD19^+^ B cells, in the spleens of the Control group, the +LPS group, and the +LPS+OT group; F–I) Relative expression levels of IL‐1β, IFN‐γ, IL‐4, and TGF‐β in the spleens of the Control group, the +LPS group and the +LPS+OT group, with the Control group levels normalized to a value of 1; J) Representative flow cytometry plots and percentage statistics of F4/80^+^CD11b^+^CD86^+^ M1‐like macrophages in the Control group, +LPS group and +LPS+OT group; K) Representative flow cytometry plots and percentage statistics of F4/80^+^CD11b^+^CD206^+^ M2‐like macrophages in the Control group, the +IL‐4 group and the +IL‐4+OT group. Two‐way ANOVA and post hoc analyses, *n* = 6 mice per group.

To explore the direct effects of OT on splenic immune cells, we co‐cultured splenic lymphocytes with LPS and OT (**Figure** **5**A). OT stimulation enhanced intracellular calcium signaling, indicating activation of cellular pathways (Figure 5B), although it did not significantly affect the proportions of CD4^+^ T cells, CD19^+^ B cells, or Foxp3^+^ Treg cells in non‐activated cultures (Figure S3, Supporting Information). However, OT increased Foxp3^+^ Treg cells and decreased CD4^+^ T and CD19^+^ B cells in LPS‐activated cultures (Figure 5C–E). RT‐qPCR showed that OT reduced LPS‐induced IL‐1β and IFN‐γ while increasing IL‐4 levels (Figure 5F–I). Using RAW264.7 cells, we found that OT counteracted IFN‐γ‐induced M1‐like polarization and enhanced IL‐4‐induced M2‐like polarization (Figure 5J,K). Collectively, these findings suggest that OT modulates splenic immune responses by selectively enhancing Foxp3^+^ Treg cell activation, suppressing CD4^+^ T and CD19^+^ B cell proliferation under inflammatory conditions, and directing macrophage polarization toward an M2‐like phenotype. These effects highlight OT's potential as a regulator of immune homeostasis in stress‐related immune dysregulation.

### 2.6 Connection from Spleen to OT Neurons of PVN

**Figure 6:**
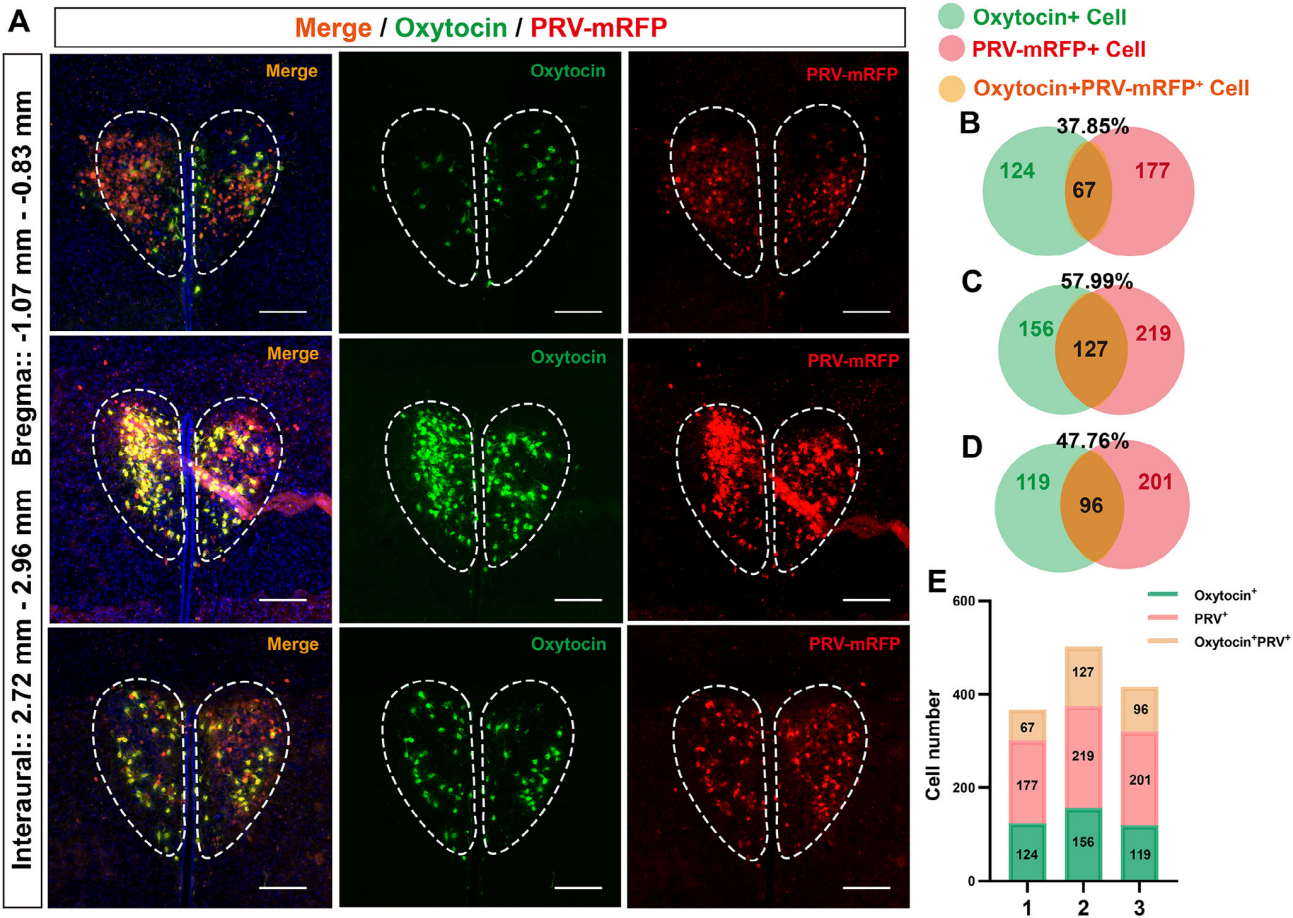
Co‐localization of PRV‐CAG‐Mrfp in the PVN with oxytocin neurons. A) After intravenous injection of the retrograde tracer virus PRV‐CAG‐Mrfp for 144 h, PVN brain sections were selected for immunofluorescent staining of oxytocin to observe the co‐localization of PVN‐mRFP^+^ neurons (red) with oxytocin neurons (green); B–D) Green is the number of OT^+^ cells (left), red is the number of PRV‐CAG‐Mrfp^+^ cells, and orange is the percent of OT^+^ cells to the PRV‐CAG‐Mrfp^+^ cells; E) Distribution of Cell Types. *n* = 3.

To establish the neural regulation of splenic immune function by chronic social stress, we employed pseudorabies virus tracing to identify the splanchnic nerve pathway to the PVN. First, we injected the PRV‐CAG‐Mrfp virus into the spleens of mice, and six days later, Mrfp signals appeared in the PVN, solitary tract nucleus, a dorsal motor nucleus of the vagus nerve, central amygdala, and so on (Figure S4, Supporting Information). Evidence supports a neural link between the PVN^CRH^ neurons and the spleen, modulating B cell and plasma cell responses.^[^ ^15^ ^]^ We propose that OT neurons may similarly modulate splenic immunity. Double immunofluorescence on PVN sections confirmed oxytocinergic neurons (**Figure** **6**A). We quantified the co‐localization of OT‐positive neurons in the PVN with neurons retrogradely labeled from the spleen using PRV‐mRFP (Figure 6B–E). Our results indicate that a subset of OT‐expressing neurons in the PVN project to the spleen. Given the increased OT levels and Treg cell proliferation observed in our chronic social stress model, we hypothesize that PVN^OT^ neurons may regulate splenic Treg cells through neuro‐immune interactions, thereby influencing immunosuppression.

### 2.7 Effects of Splenic Nerve Denervation on Behavior and Serum OT Levels in Mice with CSS

**Figure 7:**
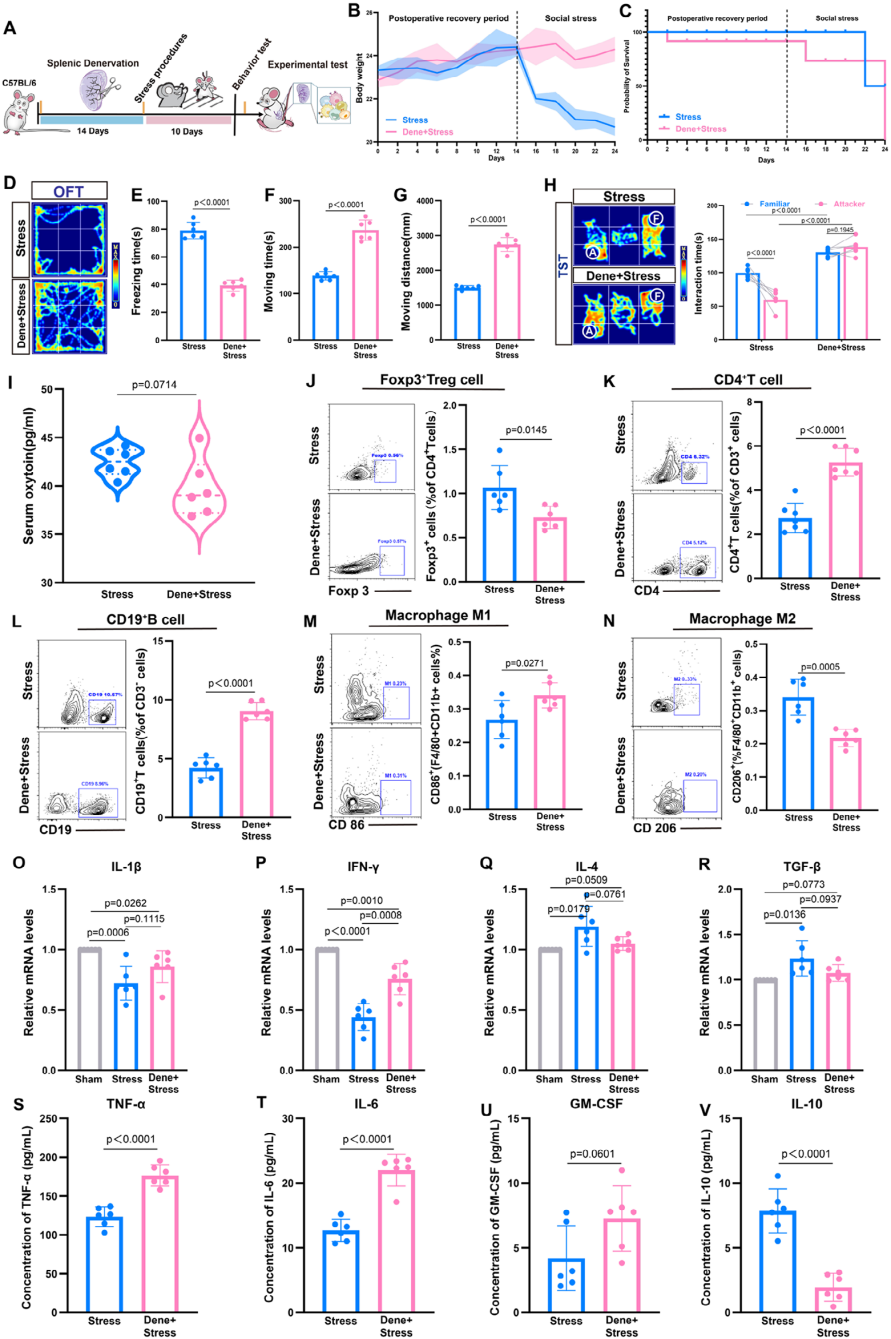
Impact of CSS in mice with denervated spleens. A) Schematic diagram of the experimental model; B) Changes in mice body weight; C) Variations in mice survival rates; D–G) Results of the OFT; (D) Activity trajectory heatmap; (E) Statistical chart of freezing time; (F) Statistical chart of moving time; (G) Statistical chart of moving distance; H) Social preference in the TST between familiar mice and attacker mice; I) Changes in serum OT levels in mice; J–N) Representative flow cytometry plots and percentage statistics of CD4^+^ T cells, CD19^+^ B cells, Foxp3^+^ Tregs, F4/80^+^CD11b^+^CD86^+^ M1‐like macrophages, and F4/80^+^CD11b^+^CD206^+^ M2‐like macrophages in the spleens of the Stress group and the Dene+Stress group; O–R) Relative expression levels of IL‐1β, IFN‐γ, IL‐4, and TGF‐β in the spleens of the Sham group, the Stress group, and the Dene+Stress group, with the Sham group levels normalized to a value of 1; S–V) The expression levels of TNF‐α, IL‐6, GM‐CSF, and IL‐10 in mouse plasma were detected by ELISA. Two‐way ANOVA and post hoc analyses, *n* = 6 mice per group.

To elucidate the role of OT neuromodulation in CSS, we modeled surgical denervation (Figure S5A, Supporting Information). Postoperative weight loss was transient but returned to baseline levels by day 14 (Figure S5B, Supporting Information), with no effect on survival (Figure S5C, Supporting Information). Immunofluorescence analysis at day 14 confirmed the absence of Th^+^ nerve fibers in the spleen (Figure S5D, Supporting Information), no significant alteration in spleen structure (Figure S5E, Supporting Information), and OT levels were comparable to control mice (Figure S5F, Supporting Information). Behavioral assessments on day 14 revealed no deficits in social functioning (Figure S5G–L, Supporting Information), and no notable changes in spleen structure compared with controls (Figure S6, Supporting Information). Subsequently, after 10 days of CSS, we evaluated social behavior and splenic immunity in mice with denervated spleens (**Figure** **7**A). Denervated mice exhibited rapid recovery of body weight post‐CSS (Figure 7B), a reduced survival rate (Figure 7C), and no significant changes in serum OT levels (Figure 7I). In the OFT, these mice showed decreased freezing time and increased movement compared to stressed controls (Figure 7D–G). They maintained normal social behavior and preferences in the TST but failed to distinguish between familiar and aggressive mice (Figure 7H), suggesting that OT neural regulation influences stress perception and recognition. These findings emphasize the importance of intact splenic nerves in the stress response and behavior, with OT potentially mediating these effects through neural pathways. To investigate the impact of OT neural regulation on the splenic immune environment following CSS, we examined changes in immune cell populations within the spleen. Compared to stressed mice, denervated mice showed increased percentages of CD4^+^ T cells and CD19^+^ B cells, while the percentage of Foxp3^+^ Treg cells was reduced (Figure 7J–L). Additionally, denervation promoted polarization of macrophages toward the F4/80^+^CD11b^+^CD86^+^ M1‐like phenotype and inhibited polarization toward the F4/80^+^CD11b^+^CD206^+^ M2‐like phenotype (Figure 7M,N). Cytokine analysis revealed elevated levels of IL‐1β and IFN‐γ and reduced levels of IL‐4 and TGF‐β (Figure 7O–R). Meanwhile, the ELISA results indicated that, compared with the stress group, after splenic denervation, TNF‐α, IL‐6, and GM‐CSF levels increased, while IL‐10 levels decreased (Figure 7S–V). These findings suggest that blocking OT neural regulation of Treg cells alters the immunosuppressive state of the spleen.

## 3 Discussion

Our study demonstrates that CSS significantly elevates serum OT levels, suppressing splenic immune responses by increasing the proportion of Foxp3^+^ Treg cells and inhibiting the numbers of CD4^+^ T and CD19^+^ B cells. Additionally, OT appears to drive macrophage polarization toward the M2‐like phenotype while inhibiting the M1‐like phenotype, thereby modulating immune responses during CSS. In addition to cellular immune changes, our results demonstrated that chronic social stress (CSS) significantly altered circulating cytokine levels. Specifically, we observed a decrease in pro‐inflammatory cytokines including IFN‐γ, IL‐1β, IL‐6, TNF‐α, and GM‐CSF, alongside an increase in the anti‐inflammatory cytokines including TGF‐β, IL‐4, IL‐10. Interestingly, the blockade of splenic denervation or OTR antagonism reversed those changes. These findings reinforce the notion that oxytocin signaling plays a central role in stress‐induced immunomodulation. This immunosuppressive effect likely prevents excessive inflammatory responses and maintains immune homeostasis. Blocking OT signaling reverses this immunosuppression but results in reduced survival rates and social memory deficits, suggesting that OT's immunosuppressive function plays a protective role in CSS. Using pseudorabies virus tracing, we confirmed that OT neurons in the PVN project to the spleen, providing preliminary anatomical evidence for OT's neural regulation of splenic immunity. These findings underscore OT's protective role during CSS and highlight its complex functions in neuro‐immune interactions mediated through neuroendocrine pathways.

Studies from other laboratories have similarly shown that stress elevates OT levels. For example, significant increases in serum OT levels have been observed under emergency conditions such as restraint or electric shocks.^[^ ^30^ ^]^ In our research, CSS also induced an increase in serum OT levels, and blocking OT signaling reduced survival rates in mice. Although previous studies have shown that high levels of glucocorticoids during chronic stress can lead to hippocampal atrophy and neurotoxicity, resulting in behavioral and physiological impairments, we did not directly investigate pathological changes in the hippocampus in the present study, and based on the available studies we hypothesize that the reduction in hippocampal volume due to chronic exposure to high levels of glucocorticoids may be associated with behavioral changes in mice.^[^ ^31^ ^]^ Furthermore, OT's role in immune modulation during CSS confirms its protective function. Blocking OT signaling heightened immune responses but decreased survival rates, possibly due to the disruption of Treg cell‐mediated immune balance, which prevents excessive immune activation and tissue damage. Treg cells, characterized by CD4, CD25, and Foxp3, suppress T/B cell proliferation, preventing excessive immune reactions. This finding is consistent with OT's roles in autoimmune diseases and cancer, where it mitigates inflammatory responses.^[^ ^32^, ^33^, ^34^, ^35^ ^]^ However, more attention needs to be paid in future studies to whether hippocampal atrophy and glucocorticoid‐related neurotoxicity are associated with decreased survival in mice. In the CSS model, OT promotes Treg cell proliferation and activation, leading to increased secretion of inhibitory cytokines such as IL‐4 and TGF‐β, while reducing CD4^+^ T and CD19^+^ B cell counts and suppressing pro‐inflammatory cytokines like IL‐1β and IFN‐γ.^[^ ^36^, ^37^, ^38^ ^]^

Our comprehensive analysis combining transcriptome profiling, flow cytometry, and cytokine assays reveals that genetic changes induced by CSS are closely linked to functional immunosuppression. Specifically, the upregulation of the chemokine receptor gene Cxcr4 enhances the proliferation of Treg cells and amplifies immunosuppression, while the downregulation of the activation‐induced cytidine deaminase gene Aicda impedes B cell differentiation and lowers antibody production. These alterations result in an increase in Treg cells and a decrease in effector T and B cells, shifting the immune system toward a suppressive state and reducing its efficacy against infections and external antigens. Our findings demonstrate that the gene expression changes due to CSS are not only statistically significant but also directly relevant to immunosuppression. This is characterized by an increase in Treg cells, a reduction in T and B cells, decreased levels of pro‐inflammatory cytokines, and inhibition of the MAPK signaling pathway, collectively fostering an immunosuppressive environment.

Foxp3 is a crucial transcription factor in Treg cells, pivotal for their differentiation and maintenance. OT, via its receptor OTR, may activate downstream signaling pathways to enhance Foxp3 expression, thereby promoting the differentiation and immunosuppressive functions of Treg cells.^[^ ^39^ ^]^ The observed increase in Foxp3 expression in our experiments supports the hypothesis that OT positively influences Treg cell differentiation. TGF‐β and IL‐4 are key cytokines that regulate Treg cell functions. TGF‐β is essential for inducing the formation of peripheral Treg (pTreg) cells, while IL‐4 plays a role in regulating their anti‐inflammatory functions. The increase in TGF‐β and IL‐4 levels induced by oxytocin suggests that oxytocin may enhance the proliferation and functional capabilities of Treg cells through these cytokines.^[^ ^40^, ^41^ ^]^ STAT3 and Smad3 are crucial signal transducers and transcriptional activators that mediate cytokine signaling across various cell types. In Treg cells, STAT3 can be activated by cytokines like IL‐6, whereas Smad3 is a pivotal mediator of TGF‐β signaling.^[^ ^42^ ^]^ With oxytocin exposure, OTR may activate these transcription factors, thereby enhancing Treg cells' responsiveness to TGF‐β and promoting the expression of key genes such as Foxp3. The observed increases in TGF‐β and IL‐4 suggest that the activation of these transcription factors is a primary mechanism by which oxytocin enhances Treg cell function. IL‐4 and IL‐10 are critical cytokines in promoting macrophage polarization toward the M2 phenotype. IL‐4 is a primary inducer of M2 polarization, activating the STAT6 pathway to enhance the expression of M2 markers.^[^ ^43^, ^44^ ^]^ IL‐10, meanwhile, serves as a powerful anti‐inflammatory agent, suppressing the production and effects of inflammatory cytokines to bolster the anti‐inflammatory functions of M2 macrophages.^[^ ^45^ ^]^ In this study, oxytocin treatment significantly elevated levels of IL‐4 and IL‐10, likely due to the activation of associated signaling pathways in macrophages through OTR, thus fostering their polarization toward the M2 type. GM‐CSF regulates the activation and plasticity of macrophages, supporting both M1 and M2 polarization depending on the context.^[^ ^46^ ^]^ The changes in GM‐CSF observed in our study also support this notion. Taken together, these data reveal that oxytocin modulates immune function via neuroimmune mechanisms involving Treg and macrophage polarization. These findings suggest that OT‐induced immunosuppression is a dynamically modulated process rather than a fixed state, and may be partially compensated by alternative neuroendocrine pathways such as arginine vasopressin (AVP).^[^ ^47^ ^]^

Behaviorally, blocking OT signaling impaired social discrimination in mice but did not affect their general social abilities. For example, in the TST, OT‐blocked mice failed to recognize aggressive mice. This aligns with studies showing that OT‐blocked mice exhibit increased irritability and aggressive behavior. Studies involving humans, cats, rats, dogs, and mice also suggest that the spleen is indeed under direct brain regulation, primarily focusing on cholinergic neurons.^[^ ^48^, ^49^, ^50^, ^51^ ^]^ Whether it is immune disorders comorbid with behavioral dysfunction or disease‐like behaviors following immune dysregulation, both underscore a tight link between behavioral changes and the immune system.^[^ ^52^, ^53^ ^]^ However, how the brain influences the immune system through changes in behavioral patterns remains largely unknown. OT is considered a crucial modulator of human affiliative behavior, with most research concentrating on its regulatory effects on social behavior. Some studies propose that OT might indirectly affect the immune system by modulating social behavior, as social interaction and support can have positive impacts on the immune system.^[^ ^54^, ^55^ ^]^ In our study, blocking OT signaling did not affect mice's social abilities but led to deficits in social discrimination, evident in the TST where mice failed to recognize aggressive mice. During the establishment of the social stress model, it was observed that mice no longer avoided social interactions with aggressive mice and even initiated attacks, possibly due to the disruption of social memory and recognition abilities by OT signaling blockade.^[^ ^55^ ^]^ Studies have also shown that OT‐blocked mice exhibit mania, irritability, and increased aggressive behavior, which might be one reason for the recovery of the spleen's immune suppression status but a decline in survival rates.

In the natural world, animals often resolve social conflicts through competitive aggressive interactions. Recent studies have shown that dysregulation of OT neurons in the PVN can lead to aggressive behavior.^[^ ^56^ ^]^ Pharmacological or genetic activation of these neurons induces changes in behavioral traits within mouse colonies. Blocking OT signaling significantly increases infanticide behavior in mice, which may suggest reflecting a disruption of OT's regulatory role in social behaviors.^[^ ^57^ ^]^ Our findings are consistent with these observations, as blocking OT signaling increased aggressive behavior and reduced survival rates in mice. The ventromedial hypothalamic nucleus (VMH), particularly its ventrolateral part (VMHvl), is a critical region for regulating aggression in mice. Activation of VMHvl neurons significantly enhances aggressive behavior, whereas their inhibition suppresses naturally occurring aggression among males.^[^ ^58^, ^59^ ^]^ OTR neurons in the VMH are implicated in regulating social and aggressive behaviors.^[^ ^60^ ^]^ Moreover, the PVN and VMH are anatomically and functionally interconnected, participating in stress responses, glucose metabolism, feeding behavior, and cardiovascular regulation.^[^ ^61^ ^]^ These regions influence each other via complex neural networks and neuroendocrine pathways. For example, the VMH receives projection fibers from the PVN, while the PVN receives reciprocal innervation from the VMH.^[^ ^62^ ^]^ The VMH also contains OT receptors, suggesting a direct neural link between these two regions.^[^ ^63^ ^]^ Studies have demonstrated that CRH neurons in the PVN regulate rapid glucose release during acute stress via the PVN‐VMH circuit.^[^ ^64^ ^]^ Building on these findings, we hypothesize that the increase in aggressive behavior observed in mice following splanchnic nerve transection may result from the disruption of sensory signal pathways from the spleen to the PVN and the loss of connection with the VMH. This aggressive behavior may stem from dysregulation of PVN‐VMH circuitry, given the VMH's critical role in aggression modulation. The PVN's OT neurons may alter social behavior and regulate the splanchnic response to CSS through direct neuromodulation, providing new insights into how the brain controls immune responses during chronic social stress. Disruptions in PVN‐VMH interactions may influence behavioral patterns through neuroendocrine pathways, but the precise mechanisms remain to be elucidated. Further investigation is necessary to clarify the role of these interactions in linking behavioral and immune responses during CSS.

While our study sheds new light on how chronic social stress affects mouse behavior and splenic immune responses, there remain key questions to be addressed. Although we have provided preliminary anatomical evidence for the neural projection from OT neurons in the PVN to the spleen, a deeper functional investigation is warranted. The study did not address the dynamic changes in other immune cell subsets. Future studies should employ advanced experimental approaches to address these questions more comprehensively.

## 4 Conclusion

This study highlights the pivotal role of OT in modulating immune responses during CSS. Elevated levels of OT promote increased Treg cell numbers and polarization of M2‐like macrophages, thereby suppressing splenic immune activity, maintaining immune homeostasis, and preventing excessive inflammation. However, blocking OT signaling, while reversing immunosuppression, leads to increased aggressive behavior, social memory deficits, and reduced survival rates, underscoring the protective nature of OT's immunomodulatory effects. We also provide preliminary evidence of a neural pathway linking OT neurons in the PVN to the spleen, revealing a direct mechanism for OT's immune regulation. However, a limitation of this study is the exclusive use of male mice and the lack of direct functional manipulation of PVN oxytocin neurons, which prevents definitive conclusions about circuit‐level causality. Future research should explore sex differences, particularly concerning whether hippocampal atrophy and glucocorticoid‐related neurotoxicity impact survival in mice. Additionally, it is crucial that future studies aim to elucidate the precise mechanisms of oxytocin to achieve a more comprehensive scientific understanding.

## 5 Experimental Section

### Animals

Male C57BL/6 mice, aged four months were utilized, which were obtained from the Experimental Animal Ethics Committee of Lanzhou University. All animal care procedures and experimental protocols were thoroughly reviewed and approved by the Committee, ensuring full compliance with ethical guidelines established by the Ministry of Science and Technology of the People's Republic of China for the care and use of laboratory animals.

### Aggressor Screening (Kunming Mice)

Male Kunming mice (4–6 months old) were singly housed for at least 7 days before screening. To assess aggression, a C57BL/6J male mouse (8–20 weeks old) was introduced into the home cage of each KM mouse for 180 s. Attack latency was recorded. Mice were screened once daily for three consecutive days. KM mice that exhibited aggressive behavior (attack latency < 60 s) in at least two of the three trials were selected as aggressors. All selected KM mice were re‐screened prior to each experimental session to ensure consistent aggression.

### Susceptibility Screening (C57BL/6J Mice)

Following 10 days of social defeat stress, social interaction (SI) testing was conducted to identify susceptible mice. Each C57BL/6J mouse was exposed to a two‐phase SI test in a rectangular arena. In phase 1 (target absent), the time spent in the interaction zone was recorded. In phase 2 (target present), a novel KM mouse was placed in a wire mesh enclosure in the interaction zone, and the time spent near the enclosure was recorded.

The SI ratio was calculated as: SI ratio = time in interaction zone (target present)/time in interaction zone (target absent). Mice with an SI ratio < 1 were classified as susceptible and used for downstream experiments. Mice with an SI ratio ≥ 1 were classified as resilient and excluded from this study.^[^ ^65^, ^66^, ^67^ ^]^

### Chronic Social Stress Procedures

Kunming mice with appropriate levels of aggression were selected based on the standardization protocol to serve as the social stressors in this experiment.^[^ ^65^, ^66^ ^]^ The average weight of the Kunming mice (40 ± 5 g) was notably higher than that of the 4‐month‐old C57BL/6 mice. The selected Kunming mice (designated as “attacker mice”) were housed individually with ad libitum access to food and water, allowing them to acclimatize to their new environment for at least 7 days before the experiment commenced. A partition was installed in the cage, dividing it into two non‐communicating sections. The Kunming mouse was placed in one section, while the C57BL/6 mouse was placed in the other. Social defeat stress was initiated 24 h after this setup. C57BL/6 mice were exposed to social aggression for 10 min each day for 10 consecutive days. On the first day, the C57BL/6 mouse was introduced as an intruder into the section housing the Kunming mouse for a 10‐min period of aggression. Following this, the C57BL/6 mouse was transferred to the opposite section of the cage, where it remained for the next 24 h. On subsequent days, the C57BL/6 mouse was rotated to a new cage with a different Kunming mouse to prevent the latter from habituating to the intruder. This rotation ensured that each social aggression session remained novel for the Kunming mice. After each 10‐min aggression session, the C57BL/6 mouse was again transferred to the opposite compartment for the remainder of the 24‐h period. Throughout the 10‐day social stress model establishment, the Kunming mice remained in their original cages, while the C57BL/6 mice were rotated between different cages with various Kunming mice to maintain experimental consistency. A schematic of the experimental procedure is provided in Figure S7 (Supporting Information).

### Splenic Denervation

Anesthesia was induced in 4‐month‐old male C57BL/6 mice using sodium pentobarbital (75 mg kg^−1^, intraperitoneally). A midline incision was made below the left rib to access the abdominal cavity, and the spleen was carefully exposed by blunt dissection using forceps. The three main vascular bundles were fully exposed. Throughout the procedure, the peritoneum and surrounding organs were protected with moist sterile dressings. Using a dissecting microscope, a cotton swab soaked in anhydrous ethanol was repeatedly applied to the exposed vascular bundles. The application was performed for 15 s per area with a 5‐s interval, repeated for a total of 10–15 times, to deplete the splenic nerve fibers accompanying the vascular bundles. Care was taken to avoid excessive dripping of anhydrous ethanol, as well as to prevent significant vascular spasm or irreversible vascular damage, which could result in splenic necrosis. For the sham surgery group, the same surgical procedure was followed, but physiological saline was used in place of anhydrous ethanol. After the surgery, allow the animals to recover for 14 days and confirm the disappearance of splenic nerve fibers by immunofluorescence.^[^ ^15^ ^]^

### PRV Retrograde Tracing from the Spleen

First, mice should be fasted for 4 h before surgery. Before starting the viral injection, anesthetize the mice with sodium pentobarbital (75 mg kg^−1^). Once the mice's vital signs were stable, secure them in a lateral recumbent position on a temperature‐controlled surgical platform. Make a left‐sided subcostal oblique incision of 1 cm to fully expose the spleen and inject the PRV virus using a stereotactic injector. Dissolve PRV‐CAG‐mRFP with RNase Free Water and draw up 5 µL of the virus using a micropipette. Under a microscope, slowly insert the glass microelectrode into the spleen to a depth of 3–5 mm and inject the virus at a constant rate (1 µL/5 min). To ensure the success of the viral injection, use a multi‐point injection method. It was important to note that after each injection point was completed, the needle should be left in place for 5–10 min. After suturing the wound, allow the mice to recover on a temperature‐controlled surgical pad until they awaken from anesthesia. On the 6th day after viral injection, collect the tissue. Fix the brain in 4% PA and dehydrate it in 20% sucrose at 4 °C for 2–3 days. Continue dehydrating in 40% sucrose for another 2–3 days before sectioning the tissue into 25 µm slices using a cryostat. After mounting the sections, collect images using a research‐grade inverted microscopic imaging system (Nikon). Identify brain regions using the Allen Mouse Brain Atlas and analyze accordingly.

### mRNA‐Sequencing Experimental Method—RNA Isolation and Library Preparation

Total RNA was isolated using TRIzol reagent (Invitrogen, CA, USA) following the manufacturer's protocol. RNA purity and concentration were determined with a NanoDrop 2000 spectrophotometer (Thermo Scientific, USA), and RNA integrity was evaluated using an Agilent 2100 Bioanalyzer (Agilent Technologies, Santa Clara, CA, USA). Libraries were prepared using the VAHTS Universal V6 RNA‐seq Library Prep Kit in accordance with the manufacturer's instructions. Transcriptome sequencing and analysis were performed by OE Biotech Co., Ltd. (Shanghai, China).

### mRNA‐Sequencing Experimental Method—mRNA‐Sequencing Analysis Process

Sequencing was performed on the Illumina NovaSeq 6000 platform, generating 150 bp paired‐end reads. Raw reads in FASTQ format were processed with fastp to remove low‐quality reads, yielding clean reads for each sample. Clean reads were aligned to the reference genome using HISAT2, and gene expression was quantified as FPKM. Read counts for each gene were obtained using HTSeq‐count. Principal component analysis (PCA) was conducted in R (v3.2.0) to evaluate the biological reproducibility of the samples.^[^ ^68^, ^69^, ^70^, ^71^ ^]^

Differential expression analysis was conducted using DESeq2. Genes with a Q value < 0.05 and a fold change > 2 or < 0.5 were identified as significantly differentially expressed genes (DEGs). Hierarchical clustering of DEGs was performed in R (v3.2.0) to visualize gene expression patterns across groups and samples. A radar plot of the top 30 DEGs, highlighting upregulated and downregulated genes, was generated using the ggradar package in R.^[^ ^72^ ^]^

Enrichment analysis of DEGs was conducted using GO, KEGG pathways, Reactome, and WikiPathways, based on the hypergeometric distribution. Significantly enriched terms were identified using R (v3.2.0). Visualizations, including bar plots, chord diagrams, and bubble plots of significant enrichment terms, were generated in R. Gene Set Enrichment Analysis (GSEA) was performed with GSEA software, using predefined gene sets. Genes were ranked by the degree of differential expression between sample groups, and enrichment of the predefined gene sets was assessed at the top or bottom of the ranking.^[^ ^73^, ^74^, ^75^ ^]^

### Behavioral Tests

All behavioral assessments were conducted in a dimly lit chamber to minimize external distractions. Mice selected for testing were allowed a 12‐h acclimation period to adapt to the environment before the commencement of the experimental procedures. During the assessments, all behavioral activities were systematically recorded using video equipment and subsequently analyzed offline with an advanced video tracking system.

### Open Field Test

To assess voluntary exploration behavior and anxiety levels, an open field test was conducted using a standard experimental paradigm. The open field environment consisted of a white wooden arena measuring 50 cm × 50 cm × 40 cm. Mice were placed at the center of the arena, and their behavior was monitored for 5 min using the TEMO OFT‐100 system. The recorded variables included the total time spent moving, the distance traveled, and the duration of freezing behavior exhibited by the mice.^[^ ^76^ ^]^

### Three‐Chamber Social Test

To evaluate social competence, preference, and memory, this study adapted the standard three‐chamber social test by incorporating aggressive mice from a chronic social stress model to examine alterations in social interactions. The experimental setup consisted of a rectangular box divided into three equal‐sized sections. The test mice were initially placed in the central section, with one side section containing a container housing a stranger mouse matched for age and sex, and the other side section left empty. In the next phase, the test mice were returned to the central section, now with both side sections occupied‐one by a stranger mouse and the other by a familiar mouse that had previously cohabited with the test mouse. The final stage involved placing the test mice in the central section with one side containing aggressive Kunming mice (acting as aggressors from the social stress model) and the other side containing familiar mice. Over a 5‐min exploration period, the behavior of the test mice toward the empty cage, stranger mice, familiar mice, and aggressor mice was recorded. Behavioral activities were tracked using the TAM system, quantifying social interaction time and movement trajectories.^[^ ^77^ ^]^

### Oxytocin Receptor Antagonist Treatment

L‐371257 (MedChemExpress, USA) was a potent competitive antagonist of the oxytocin receptor (pA2 = 8.4) with a high affinity for the receptor (Ki = 19 nm). The antagonist was administered intranasally at a dose of 300 mg kg^−1^ using a pipette to deliver diluted drops into the animal's nasal cavity, facilitating the ingestion of the compound. L‐371257 was administered within the first 15–30 min of the experiment.^[^ ^78^ ^]^

### Flow Cytometry

A single‐cell suspension of spleen cells was incubated in PBS containing 1% FBS and 5 mm EDTA for 20 min. Following incubation, the suspension was stained with the following antibodies: FITC anti‐CD3, Pacific Blue anti‐CD4, APC anti‐CD25, PE‐Cy7 anti‐CD19, PE/Cy7 anti‐CD11b, APC anti‐CD86, and FITC anti‐F4/80 (all from Abcam Plc). Intracellular staining was performed using the eBioscience Intracellular Fixation & Permeabilization Buffer Set (ThermoFisher Scientific), with eFluor anti‐CD206 (ThermoFisher Scientific) and PE anti‐Foxp3 (Abcam Plc) as the primary antibodies. Cytosolic staining was performed on ice for 40–60 min, followed by intracellular staining for 20–30 min. The stained cells were analyzed using a NovoCyte Quanteon flow cytometer, with data collection and analysis conducted using NovoExpress software. Specific gating strategies used for flow cytometry analysis are illustrated in Figure S8 (Supporting Information).

### Immunohistochemistry

Tissue specimens were fixed with 4% paraformaldehyde. After embedding the samples in the OCT compound (Servicebio), they were sectioned into 3–4 µm slices. The sections were then blocked in PBS containing 0.3% Triton X‐100 and 10% FBS at 36 °C for 1 h. Primary antibodies were diluted with an appropriate antibody diluent and incubated at 36 °C for 1 h, followed by overnight incubation at 4 °C. The primary antibodies used were anti‐tyrosine hydroxylase (Abcam Plc; 1:400), anti‐CD206 (Abcam Plc; 1:100), and anti‐oxytocin receptor (OTR) (Abcam Plc; 1:200). After primary antibody incubation, tissue sections were washed three times in PBS and incubated with secondary antibodies—AF488 donkey anti‐rabbit (Abcam Plc; 1:500) and FITC donkey anti‐goat (Abcam Plc; 1:500)—protected from light for 1 h. The sections were then washed three times in PBS, dried, and blocked. Finally, the stained tissue sections were imaged using a NIKON ECLIPSE Ti2‐E microscope, and the resulting images were analyzed.

### Measurement of Serum Oxytocin Levels by ELISA

Mouse serum was collected from the retro‐orbital sinus, as previously described. The serum samples were allowed to stand for 30 min to facilitate clotting and then centrifuged at 4000 rpm for 30 min to separate the serum. OT levels in the serum were measured using an ELISA kit (Cat# RXJ202994 m, RuiXin Biotechnology).

### Real‐Time Quantitative PCR

Total RNA was extracted from both tissues and cells according to the manufacturer's instructions using the Tissue/Cell Quick Extraction Kit (Takara). Reverse transcription of the total RNA into complementary DNA (cDNA) was performed using the PrimeScript RT Master Mix (Takara). Real‐time quantitative PCR was conducted using the Q3 Real‐time Fluorescent Quantitative PCR instrument, with GAPDH serving as the endogenous control gene. The relative expression levels of target genes were calculated using the 2^‐ΔΔCT method. Specific primers for the receptors are listed in Table S1 (Supporting Information).

### Statistical Analysis

Data were expressed as either mean ± SEM or mean ± SD and were analyzed using GraphPad Prism (version 10.2.3). Statistical comparisons between two groups were made using paired or unpaired two‐tailed Student's *t*‐test, as appropriate. For experiments involving multiple groups, data were analyzed using two‐way analysis of variance (ANOVA) followed by appropriate post‐hoc tests. RNA‐seq Data Analysis and Statistical Criteria: Differential gene expression analysis was performed using DESeq2. Genes with an absolute log₂ (fold change) > 2 and a false discovery rate (FDR)‐adjusted *p*‐value < 0.05 were considered significantly differentially expressed.

For pathway enrichment analysis, KEGG and GO analyses were conducted using the ClusterProfiler package. Enrichment was considered statistically significant when the FDR‐adjusted *p*‐value was < 0.05. GSEA was carried out using the GSEA software (v4.3). Gene sets were deemed significantly enriched when the nominal *p*‐value was < 0.05 and the FDR *q*‐value was < 0.25, in accordance with GSEA standards. These thresholds were applied uniformly across analyses to ensure the statistical robustness and biological interpretability of the results.

### Ethical Statement

All animal procedures were approved by the Institutional Animal Care and Use Committee of Lanzhou University Experimental Animal Center (Approval No. 62 000 800 000 370).

## Conflict of Interest

The authors declare no conflict of interest.

## Supporting information



Supporting Information

## Data Availability

The data that support the findings of this study are available from the corresponding author upon reasonable request.
