# Supplementary material for: Oxytocin‐Mediate Modulation of Splenic Immunosuppression in Chronic Social Stress Through Neuroendocrine Pathways
Source: Adv Sci (Weinh). 2025 Apr 26;12(25):2500849. doi: 10.1002/advs.202500849 (PMC12224936; doi:10.1002/advs.202500849)
Supplement: Supplementary file 1 — Supporting Information [file ADVS-12-2500849-s001.docx]

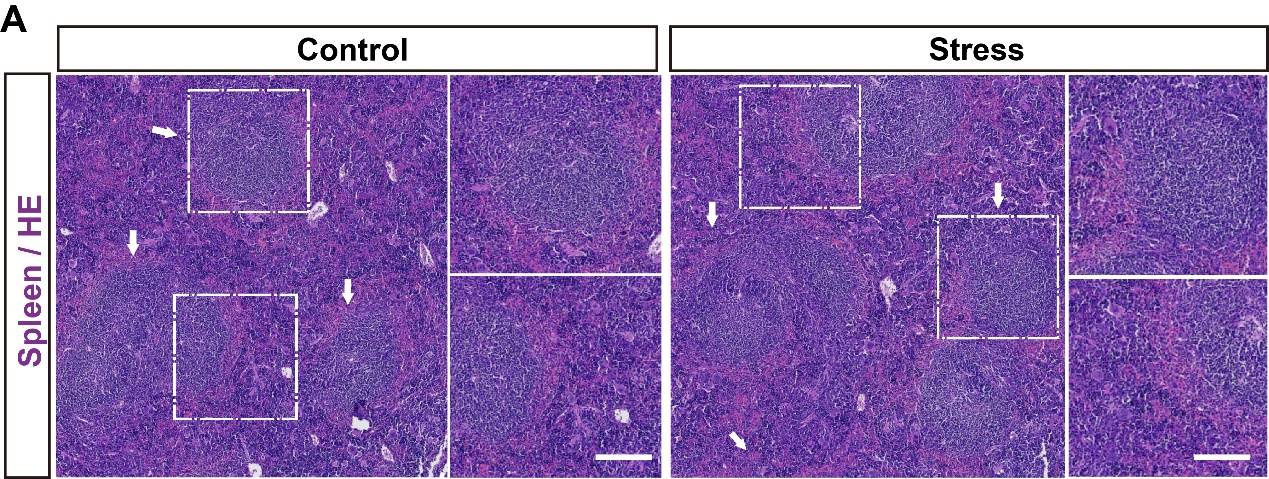


**Supplementary Figure 1.** H&E staining of spleen structure. A. Results of H&E staining of mouse spleen structure.

| 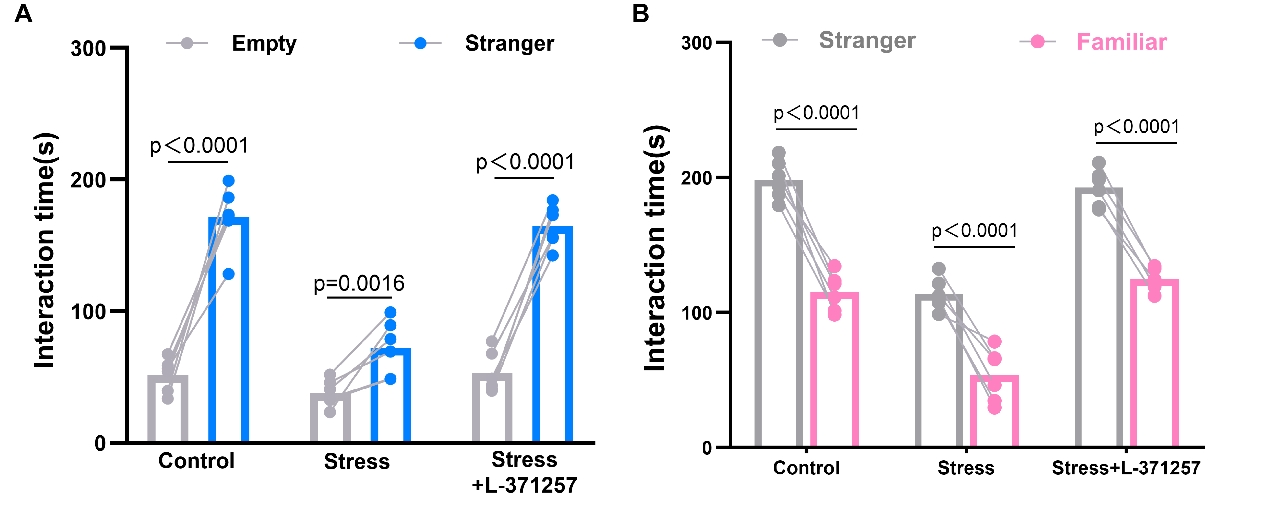 |
| --- |
|  |

**Supplementary Figure 2.** TST interaction time. A. Social preference in the TST between an empty cage and a stranger mice; B. Social preference in the TST between a stranger mice and a familiar mice; Two-way ANOVA and post hoc analyses, n = 6 mice per group.


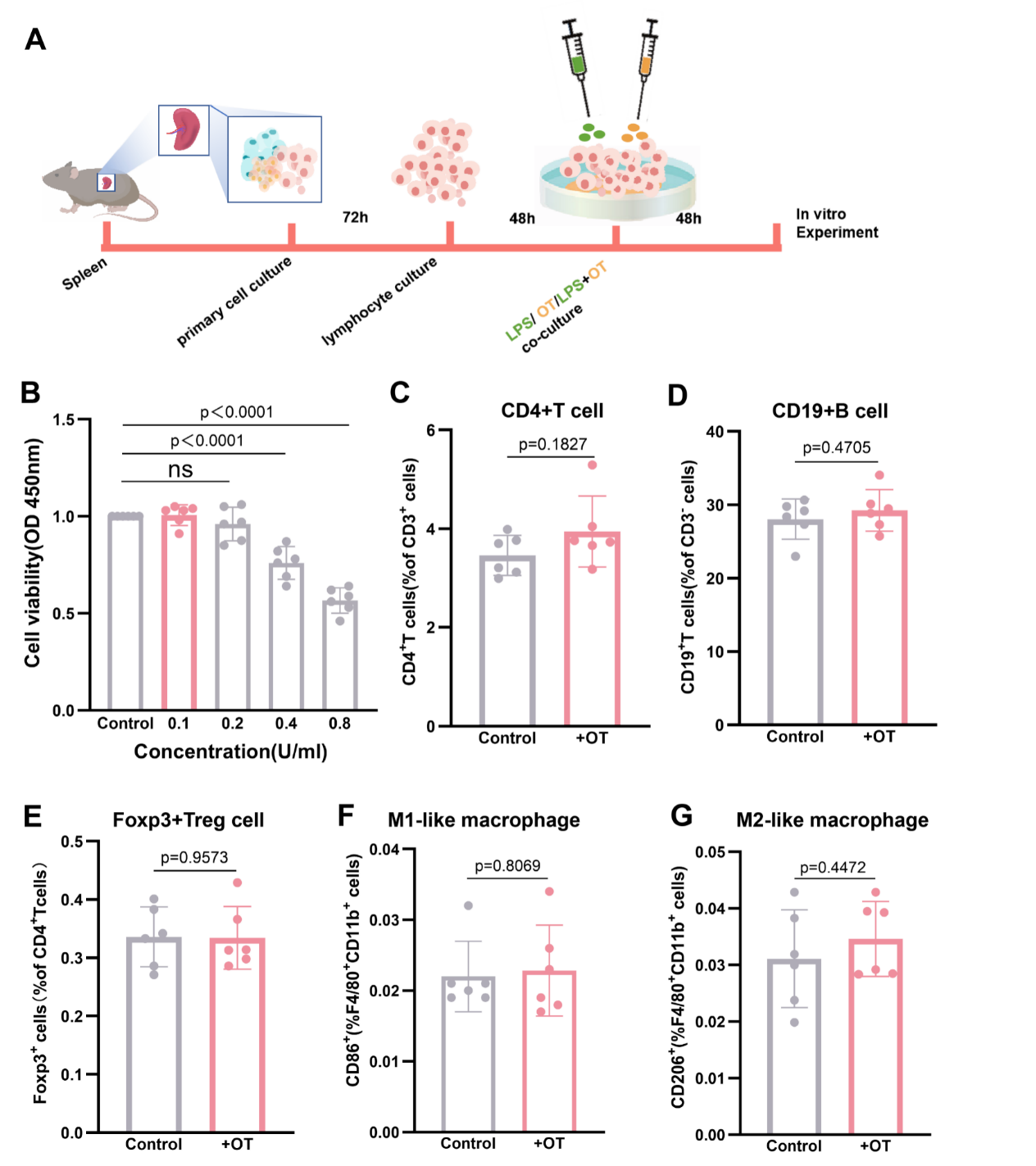


**Supplementary Figure 3.** Effect of OT on immune cells. A. Experimental pattern of splenic primary lymphocytes through B. Determination of OT concentration by CCK-8 assay; C-E Representative flow cytometry plots and percentage statistics of Foxp3^+^ Tregs, CD4^+^ T cells, CD19^+^ B cells in the spleens of the control group, and the +OT group; F-G. RAW 246.7 representative flow cytometry plots and percentage statistics of F4/80^+^CD11b^+^CD86^+^ M1 macrophages, F4/80^+^CD11b^+^CD206^+^ M2 macrophages of the control group, and the +OT group.


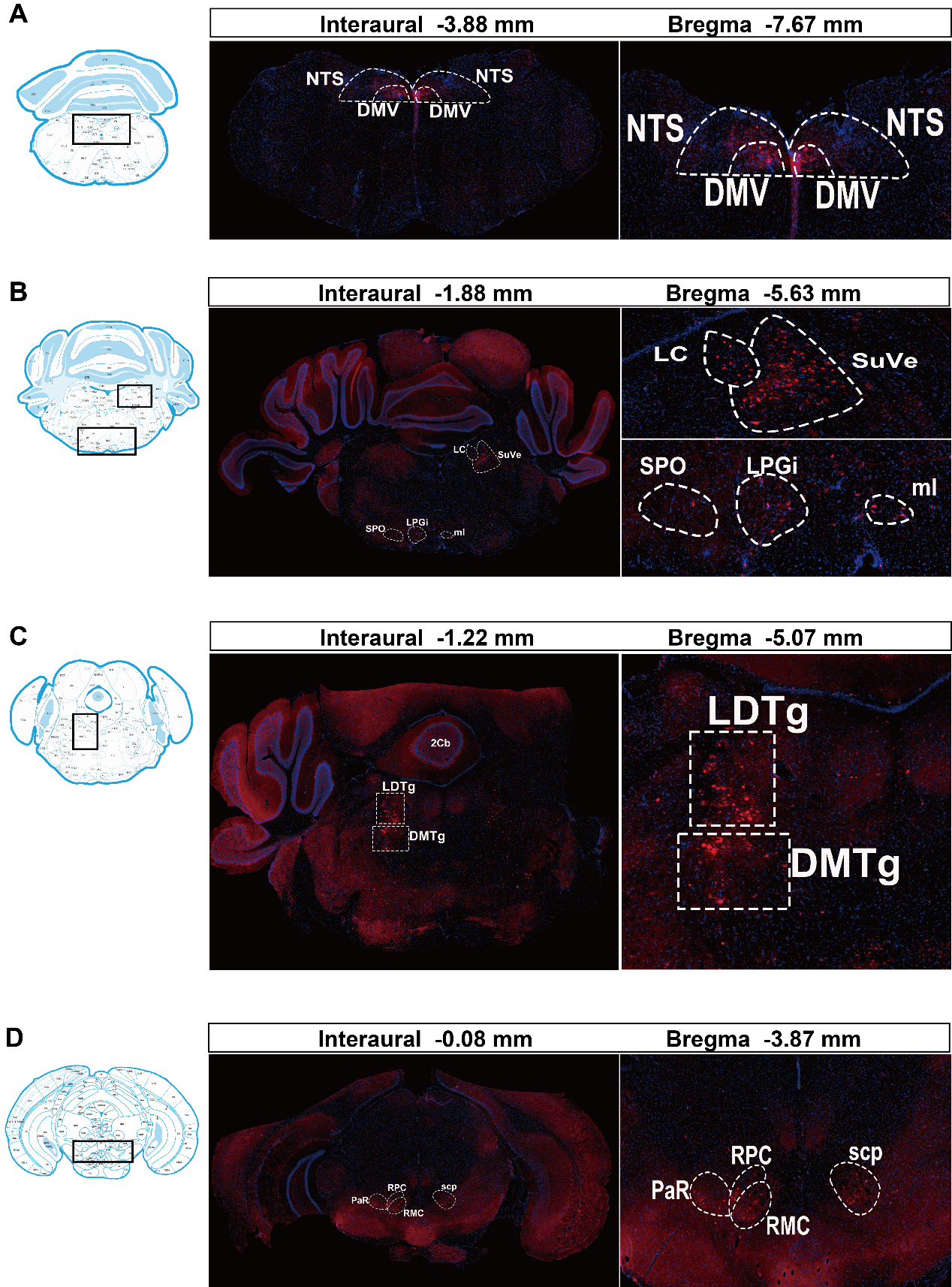


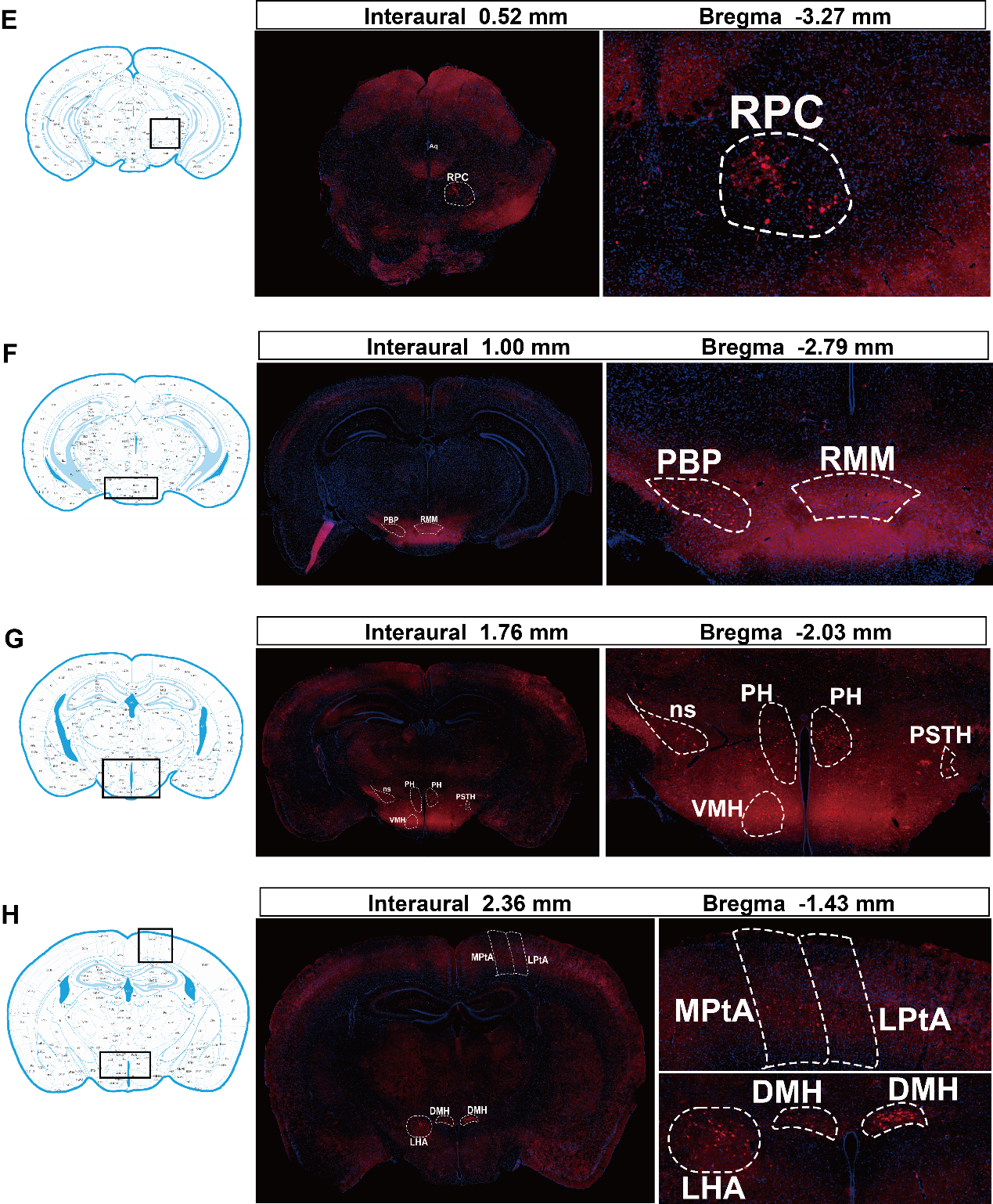


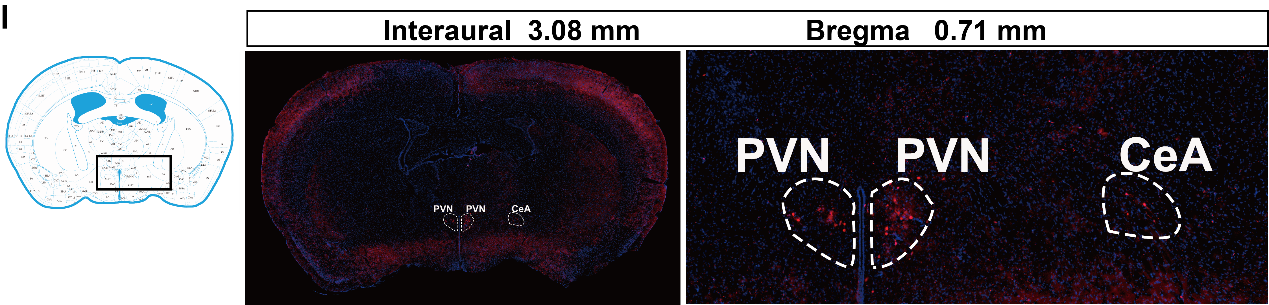


**Supplementary Figure 4.** Distribution of fluorescence 144 h after spleen injection of retrograde tracer virus PRV-CAG-mRFP. PRV-CAG-mRFP injected in the spleen can be stably expressed in nuclei such as Nucleus of the Solitary Tract (NTS), Dorsal nucleus of vagus (DMV), Locus CoeruLeus (LC), superior vestibular nucleus (ScVe), superior paraolivary nucleus (SPO), lateral paragigantocellular nucleus (LPGi), medial lemniscus (MI), red nucleus parvicellular part (RPC), parabrachial pigmented nucleus (PBP), retromammillary nucleus (RMM), posterior hypothalamic nucleus (PH), nigrostriatal bundle (Ns), parasubthalamic nucleus (PSTh), ventromedial hypothalamic nucleus (VMH), medial parietal association cortex (MPtA), lateral parietal association cortex (LPtA), Dorsomedial Hypothalamic Nucleus (DMH), lateral hypothalamic area (LHA），central amygdaloid nucleus (CeA) , PVN. The image above shows the results of one of three independent experiments.


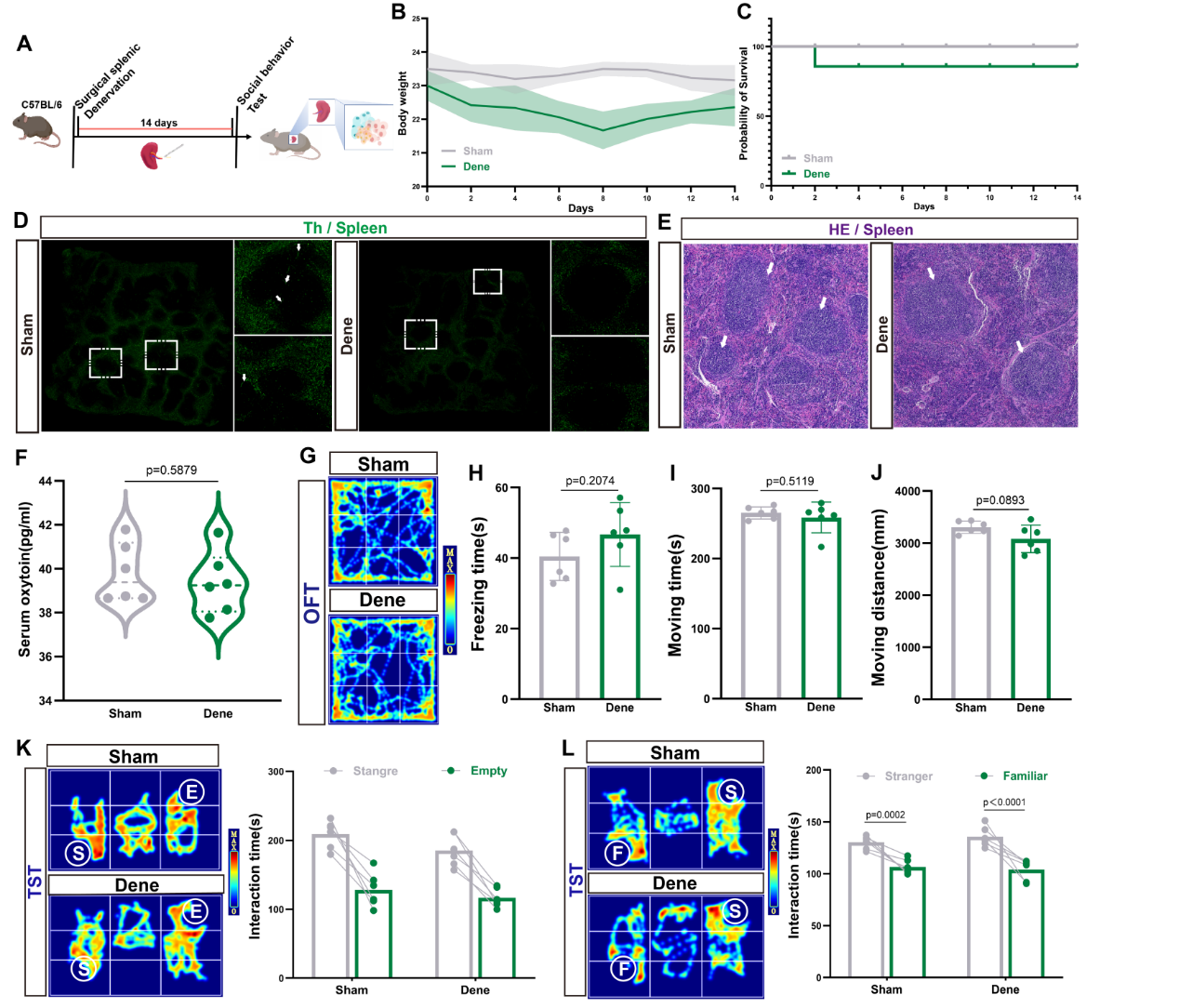


**Supplementary Figure 5.** Effects of surgical removal of the spleen in mice. A. Schematic diagram of the experimental model; B. Changes in mice body weight; C. Variations in mice survival rates; D. Images of sham-operated (left) or denervated (right) spleens 2 weeks after surgery, representative of three experiments. Green, TH staining (nerve fibres); E. H&E staining after sham surgery (left) and denervation (right) of spleen images, representative of three experiments. F. Changes in plasma OT levels in mice. G-J. Results of the OFT; G. Activity trajectory heatmap; H. Statistical chart of freezing time; I. Statistical chart of moving time; J. Statistical chart of moving distance; K. Social preference in the TST between an empty cage and a stranger mice; L. Social preference in the TST between a stranger mice and a familiar mice; K. Social preference in the TST between a familiar mice and an attacker mice; One-way ANOVA and post hoc analyses (F, H, I, J), Two-way ANOVA and post hoc analyses ( K, L), n = 6 mice per group.


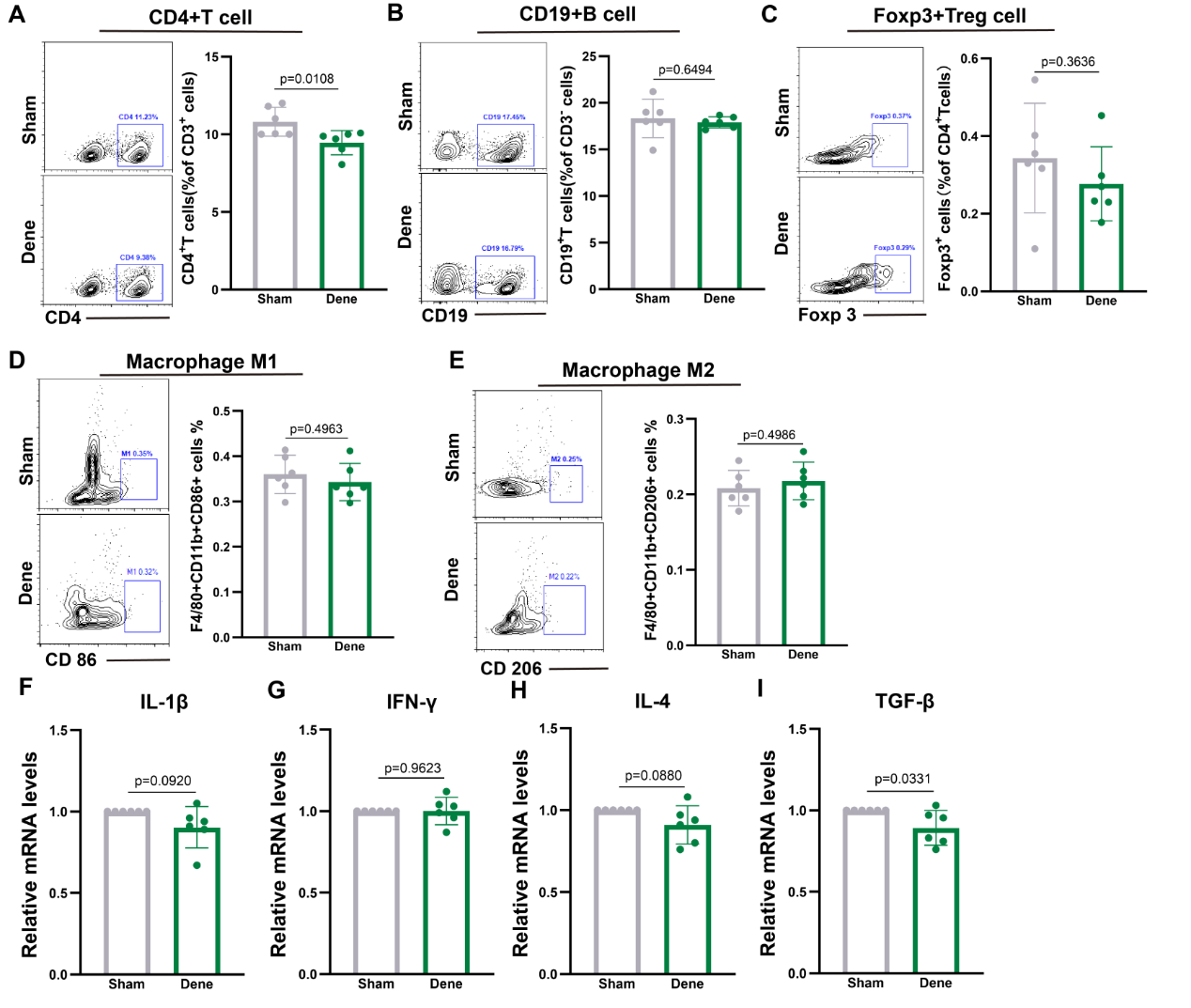


**Supplementary Figure 6.** Impact of on Splenic Immunity in mice with denervated spleens. A-E. Representative flow cytometry plots and percentage statistics of CD4^+^ T cells, CD19^+^ B cells, Foxp3^+^ Tregs, F4/80^+^CD11b^+^CD86^+^ M1 macrophages, and F4/80^+^CD11b^+^CD206^+^ M2 macrophages in the spleens of sham groups and Dene groups. F-I: Relative expression levels of IL-1β, IFN-γ, IL-4, and TGF-β in the spleens of Sham and Dene groups, with sham group levels normalized to a value of 1. One-way ANOVA and post hoc analyses, n = 6 mice per group.

**Supplementary Figure 7. Modeling of chronic social stress**


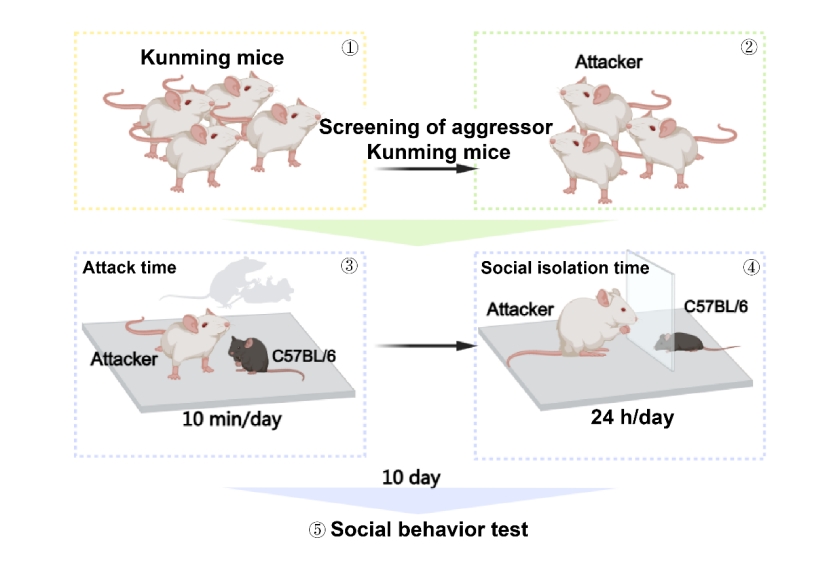


| 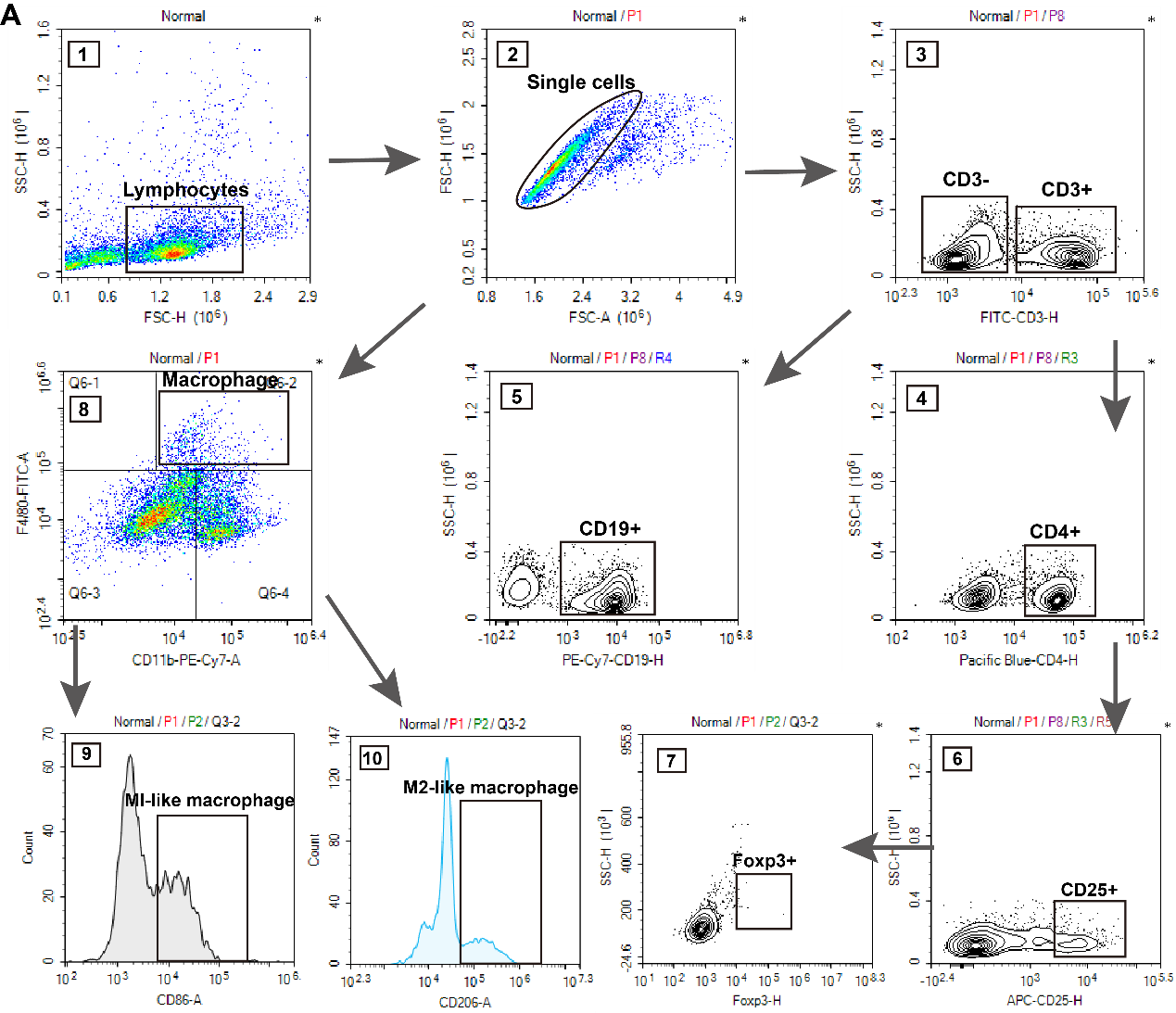 |
| --- |
| **Supplementary Figure 8.** Specific gating method |

| Gene | Forward (5’-3’) | Reverse (3’-5’) |
| --- | --- | --- |
| GAPDH  IFN-γ  TGF-β  IL-1β  IL-4 | ACTCCACTCACGGCAAATTC  CAGGCCATCAGCAACAACAT  TGACGTCACTGGAGTTGTACGG  ATA AGCCCACTCTACACCT  CTCATGGAGCTGCAGAGACTCTT | TCTCCATGGTGGTGAAGACA  GACCTGTGGGTGTTGACCT  GGTTCATGTCATGGATGGTGC  ATTGGCCCTGAAAGGAGAGA  CATTCATGGTGCAGCTTATCGA |

**Table S1.** The primer sequences
